# Extracellular vesicles in heart failure: bridging pathogenic insights, diagnostic utility, and therapeutic applications

**DOI:** 10.3389/fphar.2026.1848964

**Published:** 2026-07-31

**Authors:** Tingting Li, Jin Yang, Xinyue Ding, Zhen Qi, Zongjun Liu

**Affiliations:** 1 Department of Cardiology, Putuo Hospital, Shanghai University of Traditional Chinese Medicine, Shanghai, China; 2 Neocellmed Co., Ltd., Shanghai, China

**Keywords:** extracellular vesicle, heart failure, non-coding RNAs, biomarkers, pathogenesis, therapeutic applications

## Abstract

Heart failure (HF), the terminal stage of most cardiovascular diseases, remains a major global health burden with limited therapeutic options that do not fully achieve myocardial repair or functional recovery. Extracellular vesicles (EVs), nanoscale membrane-bound particles released by cells, have been increasingly recognized as important mediators in the pathophysiology of HF and as potential therapeutic targets. As intercellular messengers carrying bioactive molecules, including proteins, lipids, and non-coding RNAs, EVs regulate gene expression and functional states in recipient cells. This review summarizes current evidence on the roles of EVs in key pathological processes of HF, including inflammation, mitochondrial dysfunction, myocardial hypertrophy, fibrosis, apoptosis, and angiogenesis. We also discuss their potential as circulating biomarkers for the diagnosis and prognosis of HF. In addition, we describe emerging therapeutic strategies, including stem cell-derived EVs and engineered EVs as delivery platforms for therapeutic molecules. Furthermore, we discuss the pharmacological implications of EVs in current heart failure treatment paradigms, highlighting their potential role in bridging mechanistic insights with clinical therapeutic strategies. Collectively, these findings highlight the relevance of EVs in HF research and suggest directions for future investigation.

## Graphical Abstract

**Figure:**
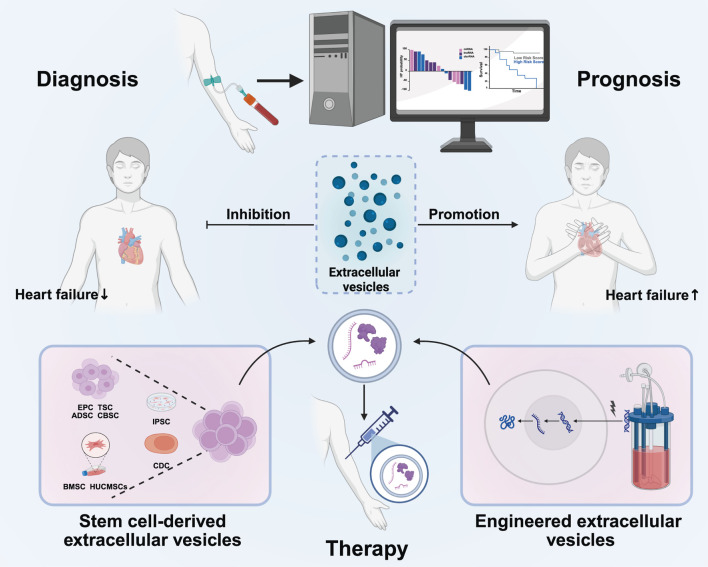

## 1 Introduction

According to the 2023 World Heart Report, cardiovascular disease (CVD) remains the leading cause of death globally, accounting for 33% of all deaths worldwide (Tsao et al., 2023). Heart failure (HF) represents the terminal stage of various cardiovascular diseases and is characterized by the heart’s inability to meet the body’s metabolic demands. Approximately 64 million individuals are currently affected worldwide, with its prevalence projected to reach 10% by 2030 (Ponikowski et al., 2016; Shahim et al., 2023). Driven by population growth and aging, the burden of HF has also increased among younger populations, placing substantial pressure on healthcare systems (Groenewegen et al., 2020; Savarese et al., 2023). The pathophysiology of HF is complex and involves inflammation, oxidative stress, mitochondrial dysfunction, apoptosis, and vascular impairment, all of which contribute to adverse cardiac remodeling, fibrosis, and progressive ventricular dysfunction (Lam et al., 2023). Despite advances in pharmacological therapies, effective strategies for myocardial repair remain lacking (Tamargo et al., 2019; Orso et al., 2022). Although non-pharmacological interventions have improved survival, long-term outcomes remain suboptimal (Sapp et al., 2024; Yafasova et al., 2024). Current therapies primarily slow disease progression, however, adult cardiomyocytes have limited regenerative capacity, and no curative strategy is currently available to reverse established myocardial damage (Tang et al., 2020). In addition, the lack of sensitive early biomarkers continues to hinder timely diagnosis and risk stratification of HF, underscoring the need for novel diagnostic approaches. According to the 2023 ESC Guidelines for the diagnosis and treatment of acute and chronic heart failure (CHF), HF diagnosis requires the presence of typical symptoms and/or signs, together with objective evidence of structural or functional cardiac abnormalities. Among these, natriuretic peptides (BNP or NT-proBNP) and echocardiography remain the cornerstone modalities for contemporary diagnosis and classification of HF (McDonagh et al., 2023). However, current diagnostic approaches remain limited in early detection, precise risk stratification, and dynamic disease monitoring. Therefore, identification and validation of novel biomarkers remain a critical priority.

Extracellular vesicles (EVs) are lipid bilayer–enclosed particles released by nearly all cell types. EVs transport a wide range of bioactive molecules, including proteins, metabolites, nucleic acids, and non-coding RNAs (ncRNAs), to recipient cells, thereby modulating cellular phenotype and function (Welsh et al., 2024). NcRNAs, including circular RNAs (circRNAs), long non-coding RNAs (lncRNAs), and microRNAs (miRNAs), are transcribed but not translated into proteins and instead regulate gene expression and disease progression at the post-transcriptional level. Among these, miRNAs represent one of the most abundant EV-associated RNA species and play a central role in gene regulation. Both circRNAs and lncRNAs are selectively enriched in EVs and can modulate miRNA activity through competitive endogenous RNA networks following intercellular transfer (Villarroya-Beltri et al., 2013). Preclinical studies have demonstrated that stem cell-derived EVs attenuate inflammation, reduce fibrosis, and promote cardiac repair (He et al., 2018). In addition, EVs can traverse biological barriers and exhibit intrinsic targeting capabilities, making them promising candidates for next-generation drug delivery systems (Ohno et al., 2013; Alvarez-Erviti et al., 2011).

Due to their lipid bilayer encapsulation, EVs-associated ncRNAs remain relatively stable in circulation and thus represent promising disease biomarkers. Specific ncRNA signatures have been associated with HF, including differential miRNA expression profiles in plasma-derived EVs from patients with chronic heart failure (CHF) compared with those with dilated cardiomyopathy (DCM) (Zhou et al., 2020; Oh et al., 2020; Zhang et al., 2023). Accordingly, EVs may represent important targets for future HF diagnosis and therapeutic intervention. In addition, EVs have been implicated in other cardiovascular diseases, including atherosclerosis, where they regulate vascular inflammation, vascular smooth muscle cell behavior, angiogenesis, and endothelial permeability (Wehbe et al., 2025).

Although previous reviews have separately discussed the roles of EVs-associated miRNAs in HF, EVs-mediated cardiac repair, and EVs-based therapeutic strategies, these works have largely focused on single mechanisms or specific applications. In contrast, this review centers on HF and integrates HF-specific mechanisms across all major pathological axes, providing a comprehensive overview of biomarkers and therapeutic pipelines. It summarizes the roles of EVs in inflammation, fibrosis, cell death, mitochondrial dysfunction, and vascular remodeling, while also integrating ncRNA-based biomarkers, engineered EVs-based therapeutic strategies, and advances in clinical translation. Importantly, this review further incorporates pharmacological implications by examining the interactions between EVs and current heart failure treatment paradigms, thereby linking mechanistic insights with therapeutic decision-making in clinical practice. By constructing a unified framework spanning mechanisms, diagnostics, and therapeutics, this review provides a comprehensive reference for the study and clinical application of EVs in HF.

## 2 EVs overview

**FIGURE 1:**
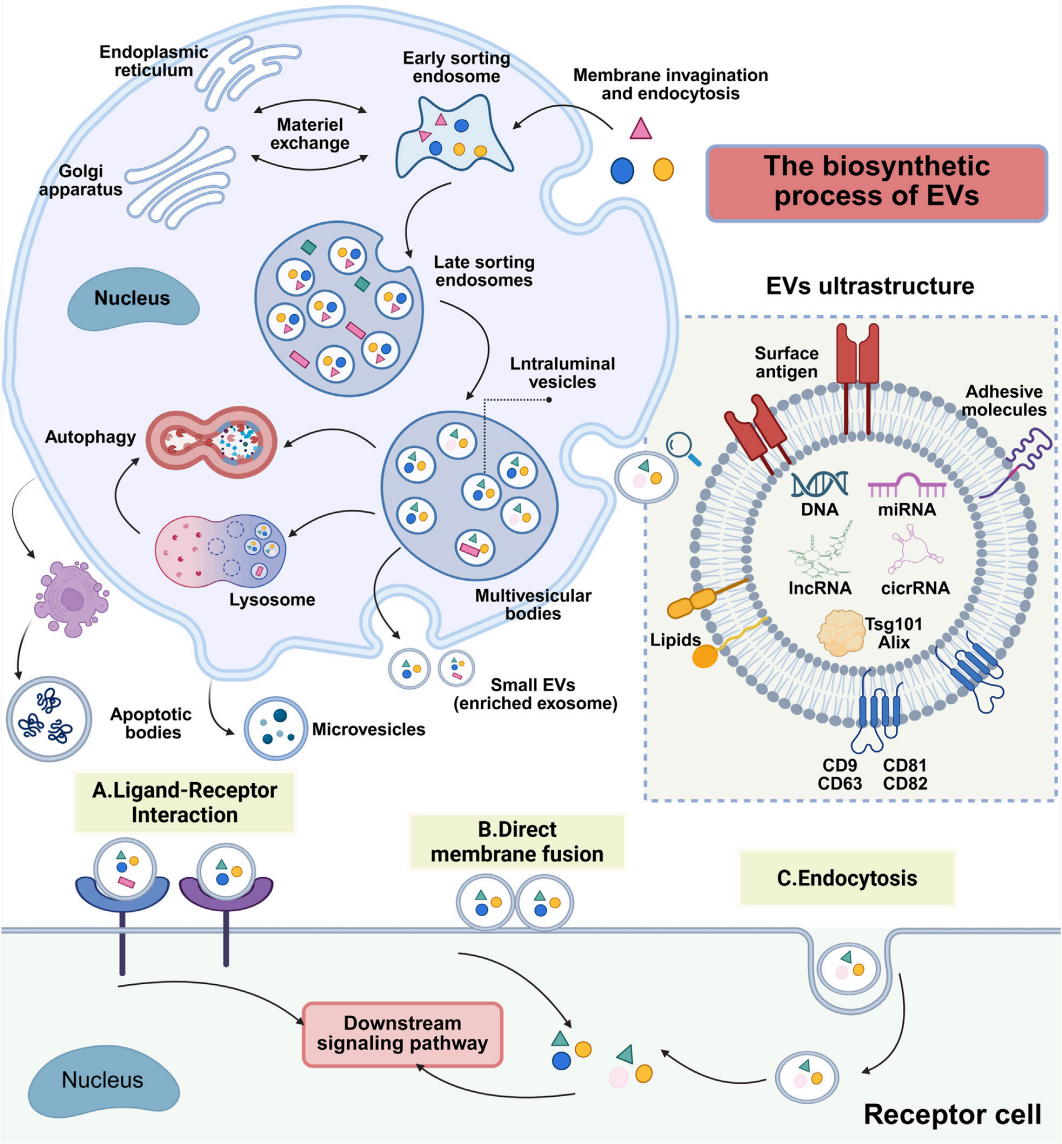
The process of EVs biogenesis and their roles in physiological and pathological processes. EVs are generated through multiple biogenetic pathways, including the endosomal pathway, direct plasma membrane budding, and cellular fragmentation during apoptosis. Once released, EVs mediate intercellular communication through three primary mechanisms: direct membrane fusion, endocytosis, and ligand–receptor interactions, thereby participating in diverse physiological and pathological processes.

**FIGURE 2:**
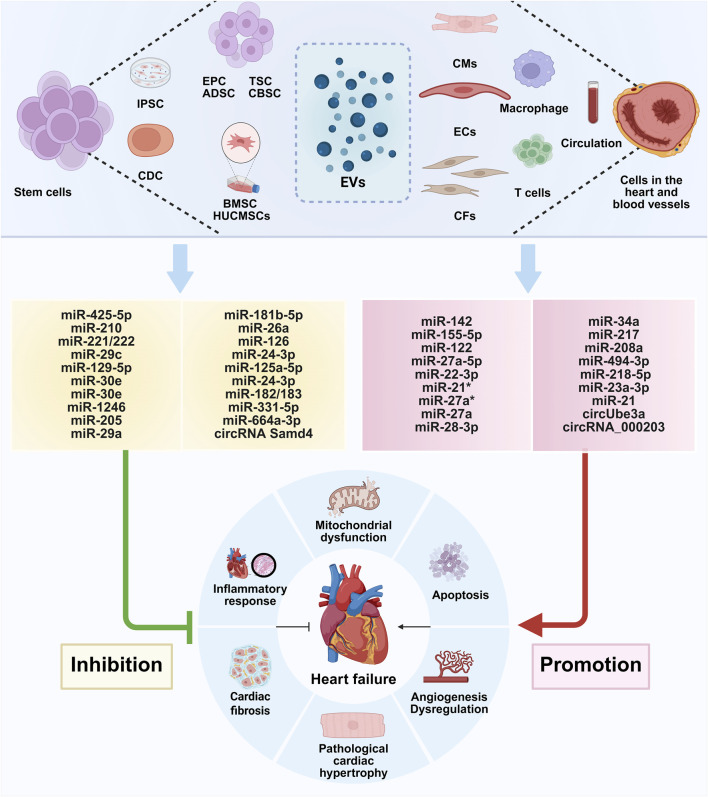
The regulatory role of EVs in the pathological progression of HF. EVs derived from various cell sources carry diverse non-coding RNAs (lncRNAs, circRNAs, and miRNAs) and regulate key pathological processes, including mitochondrial dysfunction, inflammatory response, apoptosis, cardiac fibrosis, angiogenesis dysregulation, and pathological cardiac hypertrophy, thereby contributing to either inhibition or promotion of HF progression. ADSC, Adipose-derived stem cells; BMSC, Bone marrow mesenchymal stem cells; CBSC, Cord Blood Stem Cells; CDC, Cardiosphere-derived cells; ECs, Endothelial Cells; CFs, cardiac fibroblasts; CMs,Cardiomyocytes; EPC, Endothelial progenitor cells; ESC, Embryonic stem cell; HUC-MSCs, Human umbilical cord mesenchymal stem cells; IPSC, Induced pluripotent stem cells; TSC,Trophoblast stem cell.

Almost all cell types release EVs, which are lipid bilayer–enclosed particles involved in intercellular communication. In accordance with the MISEV2023 guidelines, EVs is used as the standard term throughout this review, as most currently isolated preparations represent heterogeneous small EVs populations rather than strictly defined subtypes despite being referred to as exosomes in earlier studies (Welsh et al., 2024). EVs comprise heterogeneous lipid bilayer–enclosed particles that are generated through multiple biogenetic pathways. Among them, small EVs (commonly referred to as exosomes) are generated through the endosomal pathway and undergo a multistep biogenesis process. First, extracellular and cell-surface components, including proteins, lipids, and nucleic acids, are internalized via membrane invagination and endocytosis, leading to the formation of early endosomes. These mature into late endosomes, which undergo inward budding to generate intraluminal vesicles (ILVs) containing proteins, nucleic acids, and lipids, thereby forming multivesicular bodies (MVBs). Finally, MVBs may fuse with the plasma membrane to release ILVs into the extracellular space. In addition to endosome-derived small EVs, microvesicles are formed by direct outward budding of the plasma membrane, whereas apoptotic bodies are generated during programmed cell death through cellular fragmentation (Booth et al., 2006; Menck et al., 2017; Raposo et al., 1996). EVs are commonly enriched in tetraspanins (CD9, CD63, CD81, and CD82) and endosome-associated proteins such as TSG101 and ALIX, which are widely used for EVs characterization (Kowal et al., 2016; Romancino et al., 2013). They also carry diverse bioactive cargo, such as miRNAs, enzymes, and lipids, which can be transferred between cells to facilitate intercellular communication and exert vital functions (Jeppesen et al., 2019). Emerging evidence indicates that EVs, including exosomes when specifically characterized, participate in physiological and pathological processes across various diseases through three primary mechanisms: direct membrane fusion, endocytosis, and ligand–receptor interactions mediated by surface proteins (Parolini et al., 2009; Tian et al., 2014; Nazarenko et al., 2010) (Figure 1). The biological relevance of EVs in cardiovascular diseases is increasingly recognized, and their specific roles in HF will be discussed in the following section (Figure 2).

## 3 The regulatory role of EVs in the pathological progression of HF

### 3.1 Balancing inflammatory responses in HF by EVs

Chronic inflammation is both a contributor to HF and a key mechanism in its pathophysiology (Akhmerov and Parimon, 2022). In an experimental autoimmune myocarditis model, circulating EVs induce CD4^+^ T cell metabolic dysfunction by transferring miR-142, thereby amplifying cardiac injury through suppression of methyl-CpG binding domain protein 2 (MBD2) and suppressor of cytokine signaling 1 (SOCS1) (Sun et al., 2020). During ischemia/reperfusion (I/R)-induced myocardial injury, extracellular miR-155-5p transferred to macrophages (Mφ) activates the Janus Kinase (JAK)/signal transducer and activator of transcription 1 (STAT1) pathway, promoting M1 macrophage proliferation and exacerbating cardiac and systemic inflammation (Ge et al., 2021). Plasma-derived EVs from patients with CHF also trigger inflammatory responses via the Toll-like receptor 9 (TLR9)/nuclear factor kappa-B (NF-κB) pathway, in which mitochondrial DNA carried by EVs plays a key role in inducing inflammation (Ye et al., 2017).

In contrast, EVs derived from certain cell sources exhibit anti-inflammatory effects during cardiac injury. After I/R-induced cardiac damage, EV-associated miR-181b-5p promotes polarization of CCR2^+^ macrophages toward the M2 phenotype, facilitating endothelial cell (EC) migration and increasing vascular endothelial growth factor (VEGF) expression (Li et al., 2023). Cardiosphere-derived cell (CDC)-derived EVs carry immune-related and cardiac-related molecular cargo, serving as key mediators in immune regulation and cardiac repair; they facilitate cardioprotection through miR-181b-mediated downregulation of protein kinase C δ (PKCδ) in macrophages and miR-26a-induced sustained expression of tyrosine-protein kinase Mer (MerTK) and complement C1q subcomponent A (C1qa) following myocardial ischemic injury (López et al., 2020; de Couto et al., 2017; de Couto et al., 2019). Additionally, miR-222 from endothelial EVs and miR-126 in endothelial cells reduce intercellular adhesion molecule-1 (ICAM-1) expression, thereby decreasing monocyte adhesion and inflammatory cytokine secretion (Jansen et al., 2015; Zhang et al., 2020). A recent study demonstrated that EVs derived from IL-4–treated human THP-1 macrophages (THP1-IL4-EVs) reverse cardiac dysfunction after myocardial infarction by suppressing type I interferon signaling in circulating and myeloid cells, thereby regulating hematopoiesis, cardiac immune cell recruitment, and resident macrophage maintenance to alleviate cardiac inflammation and HF (Ng et al., 2026).

Mesenchymal stem cells (MSCs) have emerged as a promising therapeutic strategy for improving cardiac function in HF. MSC-derived EVs promote the expression of interleukin-10 (IL-10), IL-33, and IL-34 via activation of the protein phosphatase 2A (PP2A)/phosphorylated protein kinase B (p-Akt)/forkhead box O3 (Foxo3) signaling pathway in MHC-II^+^ antigen-presenting cells, thereby establishing a niche for regulatory T cells (Tregs), whose enhanced cardiac recruitment and differentiation contribute to reduced inflammation and improved cardiac repair (Zhu D. et al., 2022). Among these mechanisms, miRNAs derived from MSCs play a key role in regulating macrophage polarization. miR-182 enhances M2 macrophage polarization by targeting the TLR4 pathway, thereby reducing inflammation and infarct size in a myocardial I/R mouse model (Zhao et al., 2019). Similarly, miR-125a-5p and miR-24-3p promote M2 polarization and reduce cardiac inflammation by targeting the TRAF6/IRF5 and PLCb3-NF-κB pathways, respectively, while miR-25-3p and miR-139-3p reduce M1 macrophage numbers by suppressing STAT1 and deactivating the JAK2/STAT3 signaling pathway (Gong Z. T. et al., 2024; Zhu F. et al., 2022; Du et al., 2024; Ning et al., 2023). Similarly, EVs derived from AHR-overexpressing hUC-MSCs attenuate H/R-induced cardiomyocyte injury through AHR/NLRP3 signaling and promote anti-inflammatory immune-cell polarization (Qin Y. et al., 2026). MiR-182/183, which target the Ras p21 protein activator 1 (RASA1) axis to promote macrophages and T cells toward a pro-repair phenotypic polarization, is abundant in exosomes derived from cortical bone marrow cells, decreasing reactive oxygen species (ROS) levels and increasing oxidative phosphorylation rates to improve heart function (Yang et al., 2023).

Although emerging studies have reported that EV-associated ncRNAs and other bioactive molecules participate in inflammatory regulation in HF, these dispersed molecular mechanisms converge at the functional level. Overall, macrophage phenotypic reprogramming represents the most consistent and repeatedly observed regulatory pattern, in which multiple EV cargos promote the transition from the M1 to the M2 phenotype, thereby alleviating inflammatory responses and facilitating tissue repair. Meanwhile, the TLR/NF-κB and JAK/STAT signaling pathways constitute central hubs of inflammatory regulation, through which EVs from diverse sources exert either pro-inflammatory or anti-inflammatory effects by modulating these core cascades. In addition, increasing evidence suggests that EVs-mediated immune regulation extends beyond inflammation suppression and involves the restoration of immune homeostasis, including Treg cell expansion, MerTK/C1qa-mediated efferocytosis, and maintenance of tissue repair–associated macrophage populations.

### 3.2 Modulation of mitochondrial homeostasis in HF by EVs

Circulating EVs enriched in miR-122 contribute to cardiac dysfunction in HF by disrupting energy homeostasis and promoting maladaptive remodeling through inhibition of the mitochondrial protein Arl-2 (Wang et al., 2019). Most studies on EV-mediated regulation of mitochondrial function in HF have focused on stem cell-derived EVs. Necrostatin-1 (Nec-1), a selective inhibitor of receptor-interacting protein 1 (RIP1), suppresses myocardial contractile dysfunction by reducing ROS production through inhibition of RIP1 kinase activity and RIP1-RIP3 interaction (Zhang et al., 2018). Induced pluripotent stem cell (iPSC)-derived EVs deliver Nec-1 to target the poly (ADP-ribose) polymerase 1 (PARP1)/apoptosis-inducing factor, mitochondria-associated (AIFM1) axis, increasing ATP and superoxide dismutase levels while reducing ROS and malondialdehyde, thereby alleviating mitochondrial dysfunction in HF (Lv et al., 2024). Trophoblast stem cell (TSC)-derived EVs enhance mitochondrial fusion by upregulating Mfn2 expression, thereby improving cardiac function (Duan et al., 2023). To protect endothelial cells (ECs) from hypoxia/reoxygenation (H/R) injury, endothelial progenitor cell (EPC)-derived EVs reduce mitochondrial fragmentation, increase ATP production and mitochondrial membrane potential (MMP), and restore MFN2 and DRP1 homeostasis, with miR-210-enriched EPC-derived EVs further enhancing these effects (Ma X. et al., 2018). Mitochondrial autophagy is essential for maintaining mitochondrial quality control. Adipose-derived stem cell (ADSC)-derived EVs regulate mitochondrial autophagy and apoptosis via the miR-221/miR-222–BNIP3–MAP1LC3B–BBC3/PUMA axis, thereby alleviating particulate matter- and I/R-induced cardiac injury (Lee et al., 2025). High levels of miR-29c, which inhibits excessive autophagy via the PTEN/Akt/mTOR signaling pathway after cardiac ischemia/reperfusion injury, are also present in bone marrow mesenchymal stem cell (BMSC)-derived EVs (Li T. et al., 2020).

Although emerging studies have reported that EV-associated ncRNAs and other bioactive cargos participate in mitochondrial regulation in HF, these mechanisms largely converge on several interconnected core processes. Mitochondrial quality control is currently the most prominent regulatory axis, encompassing mitochondrial dynamics, mitophagy, and mitochondrial biogenesis. Multiple molecules act on key nodes such as Mfn2, Drp1, PINK1, BNIP3, and AMPK to maintain mitochondrial structural integrity and functional homeostasis, whereas reductions in ROS, restoration of ATP production, and improvement of calcium homeostasis represent downstream functional outcomes of these regulatory processes. Overall, mitochondrial quality control not only underlies the majority of reported regulatory mechanisms but also intersects closely with oxidative stress and metabolic remodeling, suggesting that it may serve as a central hub linking mitochondrial structural integrity with functional adaptation.

### 3.3 Control of cardiomyocyte apoptosis in HF by EVs

EVs-associated miRNAs and ncRNAs have been implicated in the regulation of apoptosis in HF. EV-associated miR-22-3p downregulates its target gene FURIN, thereby inhibiting cellular activity and promoting apoptosis, which contributes to increased HF risk (Lu et al., 2023). EVs-derived lncRNA AK139128, enriched in hypoxic cardiomyocytes, promotes apoptosis and inhibits proliferation, migration, and invasion of cardiac fibroblasts (CFs), offering insights into hypoxia-induced HF (Wang and Zhang, 2020). Circular RNA CDR1as acts as a miRNA sponge to regulate gene expression, targeting the miR-135a/heme oxygenase 1 (HMOX1) and miR-135b/HMOX1 signaling axes to modulate proliferation and apoptosis in human cardiomyocytes, thereby participating in HF pathogenesis (Chen C. et al., 2020).

In contrast, EVs-associated ncRNAs also play a crucial role in cardioprotection. Adipose-derived stem cell (ADSC)-derived EVs increase ATP content and improve ejection fraction, thereby enhancing cardiac function while reducing serum BNP and ANP levels. Their anti-apoptotic effects are mediated by modulation of pro- and anti-apoptotic proteins in cardiac tissue, with suppression of Bax, caspase-3, and p53 as key mediators (Wang L. et al., 2023). MSC-derived EVs carrying miR-129-5p target TRAF3 and downstream NF-κB signaling to alleviate ventricular dysfunction, suppress oxidative stress, and reduce apoptosis in HF mouse cardiomyocytes, an effect attenuated by miR-129-5p inhibition (Yan et al., 2022). EVs derived from miR-30e-overexpressing bone marrow MSCs suppress lectin-like oxidized low-density lipoprotein receptor 1 (LOX1) expression, thereby downregulating NF-κB p65/caspase-9 signaling to inhibit cardiomyocyte apoptosis and fibrosis, improving post-MI HF (Pu et al., 2021). Additionally, BMSC-derived EVs containing lncRNA GAS5 activate the ULK3/Hippo pathway in HF rat and hypoxic cell models, inhibiting ferroptosis, reducing oxidative stress, and attenuating apoptosis to alleviate HF (Ren and Zhao, 2024). EVs derived from human endometrial mesenchymal stem cells incorporated into polypyrrole–chitosan conductive hydrogels exhibit broad anti-apoptotic effects and contribute to HF prevention via activation of the EGF/PI3K/AKT signaling pathway (Yan et al., 2024).

Cell death regulation in HF is not limited to classical apoptotic pathways. Although EV-associated cargos act on diverse molecular targets, most studies have focused on key pro-survival signaling pathways, including PI3K/AKT, NF-κB, Hippo, and HMOX1, through which they exert cardioprotective effects by modulating oxidative stress, ferroptosis, and copper homeostasis. Notably, these upstream regulatory events ultimately converge on common effector processes, including restoration of the Bax/Bcl-2 balance and inhibition of the caspase cascade, thereby reducing cardiomyocyte loss.

### 3.4 Shaping angiogenesis in HF by EVs

The role of EVs and their miRNAs in angiogenesis has been increasingly highlighted in recent studies. EVs from patients with HF exhibit a reduced capacity to stimulate endothelial tube formation and cardiomyocyte proliferation *in vitro* compared with EVs from healthy donors. Mechanistic studies have shown that dysregulation of miR-21-5p fails to suppress PTEN, thereby enhancing AKT phosphorylation in recipient cells and diminishing EV-mediated cardiac repair (Qiao et al., 2019). In the MI microenvironment, EV-associated miR-155 derived from pro-inflammatory M1-like macrophages is transferred to endothelial cells (ECs), where it targets multiple genes to inhibit the Sirtuin 1 (SIRT1)/AMP-activated protein kinase α2 (AMPKα2)/endothelial nitric oxide synthase (eNOS) pathway and the Rac family small GTPase 1 (RAC1)–p21-activated kinase 2 (PAK2) signaling axis, thereby reducing EC angiogenic capacity and exacerbating myocardial injury (Liu et al., 2020). Activated CFs secrete EVs enriched in miR-200a-3p, which induce endothelial dysfunction by targeting the E26 transformation-specific sequence factor-1 (ETS1)/VEGF-A signaling axis, thereby mediating crosstalk with cardiac ECs (Ranjan et al., 2021). The composition and biological activity of EVs are primarily determined by their cellular origin. In N-terminal prolactin fragment (16 kDa)-induced cardiomyopathy, EC-derived EVs enriched in miR-146a attenuate EC proliferation by downregulating NRAS, thereby inhibiting microvascular regeneration and worsening systolic and diastolic function (Halkein et al., 2013). However, EVs derived from CDCs carrying miR-146a exhibit pro-angiogenic effects and significantly improve HF after acute AMI (Ibrahim et al., 2014). These inconsistent effects of miR-146a may be associated with different stages of HF.

Notably, EVs also promote angiogenesis. Human umbilical cord mesenchymal stem cell (hUC-MSC)-derived EVs target miR-1246 and PRSS23 (serine protease 23) and inhibit Snail/α-SMA signaling, thereby enhancing myocardial angiogenesis, mitigating hypoxia-induced myocardial injury, and protecting against HF (Wang Z. et al., 2021). Embryonic stem cell (ESC)-derived EVs alleviate stress-induced HF by promoting myocardial angiogenesis, with fibroblast growth factor-2 (FGF2) playing a crucial role (Pang et al., 2021). In a mouse MI model, intravenous infusion of ADSC-derived exosomes promotes angiogenesis and enhances microvascular EC proliferation, with exosomal miR-205 serving as a key mediator in ADSC-endothelial cell crosstalk (Wang T. et al., 2023). Recent studies demonstrate that mouse serum EVs derived from Huoxue Yiqi Formula (HXYQR) treatment contain active pharmaceutical components and promote angiogenesis in human umbilical vein endothelial cells (HUVECs) under hypoxic conditions via activation of the p38/MAPK–MAPKAPK2/heat shock protein 27 (HSP27), focal adhesion kinase (FAK), and hypoxia-inducible factor 1α (HIF-1α)/VEGF pathways (Gao et al., 2025).

Angiogenic regulation exhibits a clear bidirectional pattern. Current studies show that EVs from different cellular sources promote endothelial cell proliferation, migration, and microvascular formation through canonical pathways such as VEGF, FGF2, Akt, and HIF-1α, whereas EVs derived from inflammatory cells, activated fibroblasts, and stressed cardiomyocytes more often exert inhibitory effects on angiogenesis. Notably, the same miRNA may exert opposing functions depending on its EV source. For example, miR-146a can either promote or inhibit angiogenesis, suggesting that its biological effects are jointly influenced by cellular origin and the pathological microenvironment. Therefore, restoring angiogenic capacity in injured myocardium and coordinating the regulatory effects of EVs from different cellular sources on vascular remodeling may represent an important direction for improving therapeutic outcomes in the future.

### 3.5 Regulation of pathological cardiac hypertrophy in HF by EVs

CFs serve as sensitive transducers of hypertrophy-promoting signals mediated by EVs. Early studies revealed that CFs secrete EVs enriched with miRNA passenger strands (“star” miRNAs). Notably, miR-21-3p (miR-21*) enters cardiomyocytes and targets PDZ and LIM domain 5 (PDLIM5) and sorbin and SH3 domain-containing protein 2 (SORBS2), triggering ventricular hypertrophy in a pressure overload model, an effect alleviated by pharmacological inhibition of miR-21* (Bang et al., 2014). Similarly, miR-27a* in fibroblast-derived EVs inhibits PDLIM5 translation in cardiomyocytes, promoting cardiac hypertrophy–associated gene expression (Tian et al., 2020). CFs-derived iPSC EVs exhibit reduced miR-22 expression compared with dermal fibroblast-derived iPSC EVs (DF-iPSC EVs), reflecting a loss of hypertrophic disease memory and positioning them as a promising biological resource for future HF therapies (Kurtzwald-Josefson et al., 2020). Sulforaphane (SFN), a cardioprotective isothiocyanate, enhances the release of anti-remodeling EVs from fibroblasts that target cardiomyocytes, reducing myocardial hypertrophy and scar size and improving contractility to prevent HF onset, effects associated with enhanced histone acetylation in stressed cardiomyocytes (Papini et al., 2023). Ang II also triggers cardiomyocyte hypertrophy and activates CFs via EV-mediated Shh/N-Shh/Gli1 signaling (Wang C. et al., 2024). Tipifarnib, a biosynthetic inhibitor of EV release, exerts cardioprotective effects in pressure-overloaded HF models by suppressing cardiomyocyte hypertrophy, as reflected by reduced cardiomyocyte cross-sectional area and decreased expression of hypertrophy-associated fetal genes ANP, BNP, and MYH7, correlating with suppression of EV biogenesis-related proteins Rab27a, nSMase2, and Alix (Mallaredy et al., 2024). Oxidative stress and ROS contribute to cardiac hypertrophy, with Kelch-like ECH-associated protein 1–nuclear factor erythroid 2-related factor 2 (KEAP1–NRF2) serving as the master transcriptional axis of antioxidant defense. In a rat model of CHF, the left ventricle shows elevated expression of miR-27a, miR-28-3p, and miR-34a, packaged into EVs secreted by TNF-α-stimulated cardiomyocytes and fibroblasts, thereby inhibiting NRF2 translation and antioxidant gene expression, a mechanism driving cardiac hypertrophy in ischemic HF (Tian et al., 2018). EVs-associated miR-217 is enriched in CHF hearts, and its direct target PTEN participates in cardiac hypertrophy and fibrosis (Nie et al., 2018). In Ang II-treated neonatal mouse ventricular cardiomyocytes (NMVCs) and Ang II-injected cardiomyocyte-specific circRNA_000203 transgenic mice (Tg-circ203), circRNA_000203 expression is markedly increased, elevating Gata4 expression and exacerbating cardiac hypertrophy by competitively inhibiting miR-26b-5p and miR-140-3p binding to the Gata4 3′-UTRs, while also modulating NF-κB signaling (Li H. et al., 2020). Conversely, bone marrow MSC-derived EVs reduce hypertrophy markers ANP and β-MHC, with miR-29a emerging as a critical mediator of cardioprotection under pressure overload (Chen F. et al., 2020). MSC-derived EVs inhibit cardiomyocyte hypertrophy by inactivating the Hippo–YAP pathway, thereby attenuating apoptosis and inflammatory responses (Ren et al., 2023).

Cardiac hypertrophy is not driven solely by cardiomyocyte-intrinsic mechanisms but is jointly regulated by intercellular signaling networks. In particular, EV-mediated communication between cardiac fibroblasts and cardiomyocytes is highly active, with multiple fibroblast-derived miRNAs directly regulating hypertrophy-associated gene expression in cardiomyocytes and promoting pathological remodeling. Meanwhile, oxidative stress–related pathways represent a recurrent regulatory node, in which multiple miRNAs suppress NRF2 and its downstream antioxidant network, leading to ROS accumulation and amplification of hypertrophic signaling. Although upstream regulatory molecules and cellular sources vary, most pro-remodeling effects ultimately converge on canonical hypertrophic signaling networks, including PI3K/AKT and MAPK pathways, collectively driving reactivation of fetal gene programs such as ANP, BNP, and β-MHC. In contrast, EVs derived from bone marrow MSCs and MSCs predominantly exert anti-remodeling effects by inhibiting Hippo–YAP signaling, alleviating oxidative stress, and improving cellular survival. Therefore, EVs-mediated regulation of cardiac hypertrophy exhibits marked intercellular communication dependence and oxidative stress–driven characteristics, with the final phenotype determined by EVs origin and the pathological microenvironment.

### 3.6 Regulation of cardiac fibrosis in HF by EVs

EVs derived from various cell types actively participate in the pathogenesis of cardiac fibrosis, a hallmark of HF progression. Among the pro-fibrotic mediators, cardiomyocyte-derived EVs-associated miR-208a targets dual-specificity tyrosine phosphorylation-regulated kinase 2 (Dyrk2) and promotes nuclear factor of activated T cells (NFAT) nuclear translocation, driving CFs proliferation and myofibroblast differentiation (Yang et al., 2018). Under mechanical stress or pressure overload, cardiomyocyte-derived EV-associated miR-494-3p is transferred to CFs, where it inhibits phosphatase and tensin homolog (PTEN) and activates AKT, ERK, and SMAD2/3 phosphorylation to induce fibrosis. Peli1 facilitates EV-mediated transport of miR-494-3p via NF-κB and activator protein 1 (AP-1) signaling (Tang et al., 2023). In advanced familial DCM, EVs-associated miR-218-5p promotes fibrosis by suppressing tumor necrosis factor-α-induced protein 3 (TNFAIP3) and activating transforming growth factor-β (TGF-β) signaling (Fu et al., 2023). Beyond the heart, dysfunctional adipose tissue contributes to cardiac fibrosis through EV-mediated crosstalk. Adipocyte-derived EVs-associated miR-23a-3p targets Ras-related protein 1 (RAP1) in fibroblasts, enhancing collagen deposition (Su et al., 2022). Immune cells also participate in this process. Activated CD4^+^ T cell–derived EVs deliver miR-142-3p to inhibit adenomatous polyposis coli (APC)–WNT signaling–mediated myofibroblast activation (Cai et al., 2020). Macrophage-derived EVs carrying miR-21 promote fibroblast-to-myofibroblast transition under pressure overload (Hinkel et al., 2020; Ramanujam et al., 2021). After MI, M2 macrophage-derived EVs-associated circUbe3a targets the miR-138-5p/RhoC axis to aggravate fibrosis (Wang Y. et al., 2021). Serum EVs-associated miR-320a from patients with chronic HF activates the phosphatidylinositol 3-kinase catalytic subunit alpha (PIK3CA)/AKT/mTOR pathway to drive fibrosis (Wang Q. G. et al., 2021). EVs secretion itself is modulated by βIV-spectrin, and its deficiency alters CF behavior via signal transducer and activator of transcription 3 (STAT3) signaling (Nassal et al., 2023).

In contrast, a growing body of evidence highlights the anti-fibrotic potential of EVs. Cardiac EVs-associated miR-331-5p suppresses myofibroblast activation by downregulating homeobox C8 (HOXC8), positioning it as a potential therapeutic target for tipifarnib (Mallaredy et al., 2024). Recent studies have shown that EVs-associated miR-664a-3p derived from small EVs in the plasma of young healthy males suppresses the TGF-β/SMAD4 signaling pathway by directly binding to the 3′-untranslated region of SMAD4 mRNA, thereby protecting mouse hearts from fibrosis, although its role in human cardiac fibrosis remains unclear (Wang et al., 2025). Stem cell-derived EVs have emerged as particularly promising candidates. Human cortical osteoblast-derived EVs reduce fibroblast activation in association with decreased small nucleolar RNA (snoRNA) levels (Schena et al., 2021). MSC-derived EVs inhibit fibrosis via platelet-derived growth factor receptor-β (PDGFR-β) signaling (Wang X. et al., 2021). Moreover, EVs-associated circ_0036176 suppresses cardiac fibroblast proliferation by generating the Myo9a-208 protein and inhibiting the cyclin/Rb pathway (Guo et al., 2022).

Cardiac fibrosis–related studies exhibit a relatively clear directional pattern, with pathological effects ultimately converging on the common endpoint of aberrant fibroblast activation. Most EVs derived from injured myocardium, inflammatory cells, or the pathological microenvironment display pro-fibrotic effects by regulating key signaling networks such as TGF-β/SMAD, PI3K/AKT/mTOR, WNT/β-catenin, and RhoC, thereby promoting fibroblast activation, myofibroblast differentiation, and extracellular matrix deposition. In contrast, stem cell-derived EVs and certain protective non-coding RNAs exert anti-fibrotic effects by inhibiting TGF-β/SMAD signaling, maintaining miR-331-5p/HOXC8 homeostasis, or modulating PDGFR-β–related pathways. Given that established fibrosis is often progressive and has limited reversibility, therapeutic interventions targeting these key molecular axes may provide new strategies for delaying HF progression.

### 3.7 Overall mechanistic insights

Currently, studies on the mechanisms of EVs in HF primarily focus on specific EV cargos and their individual biological effects, often categorizing them into independent processes such as anti-inflammatory, anti-apoptotic, anti-fibrotic, or pro-angiogenic functions. However, accumulating evidence indicates that EVs-mediated biological effects are inherently multi-targeted and multi-layered. Although EVs from different cellular sources carry distinct functional molecules, their downstream regulatory outcomes frequently converge on several key cellular processes and signaling networks, including canonical pathways such as PI3K/AKT, TGF-β/SMAD, NF-κB, SIRT1/Nrf2, and VEGF. Similar signaling convergence has also been observed in non-cardiac diseases, where EVs-associated miRNAs regulate vascular permeability through the PI3K/AKT pathway (Li L. et al., 2026). The bioactive molecules carried by the same EVs can act on different recipient cells and simultaneously influence multiple pathological processes, thereby collectively contributing to the progression of HF. For example, MSC-derived EVs carrying miR-21a in an ischemia–reperfusion injury model can attenuate inflammatory responses and cell death by regulating PTEN-related signaling. Whereas macrophage-derived EVs containing miR-21 in a pressure overload–induced cardiac remodeling model are associated with M1 polarization, fibroblast activation, and progression of fibrosis (Hinkel et al., 2020; Ramanujam et al., 2021; Wei et al., 2021). Similarly, miR-146a in endothelial cell–derived EVs can promote microvascular regeneration by inhibiting NRAS, whereas in CDC-derived EVs it is associated with angiogenesis and improvement of cardiac function (Halkein et al., 2013; Ibrahim et al., 2014). The effects of EVs depend not only on their bioactive components, but also closely on the cell of origin, target cell types, and disease context. Therefore, it is difficult to comprehensively characterize their roles in HF simply as “protective” or “deleterious” factors. Recent studies have further revealed that EV-mediated biological effects are not limited to the local cardiac microenvironment. Circulating EVs from patients with chronic kidney disease and from animal models can carry kidney-derived miRNAs that directly damage cardiomyocytes and promote HF progression, suggesting that EV-mediated inter-organ communication may represent an important mechanism driving cardiac pathological remodeling (Li X. et al., 2026). In addition, exercise-induced EV miRNAs may also regulate cardioprotective pathways through long-range signaling among skeletal muscle, adipose tissue, and the myocardium, such as the PI3K/Akt and NF-κB signaling networks, thereby achieving systemic cardioprotection (Li Y. et al., 2026).

Most mechanistic studies are currently based primarily on *in vitro* cell experiments and rodent models, with study endpoints largely focusing on improvements in cardiac function, histological changes, and validation of single signaling pathways. In contrast, evidence from large animal models, validation using clinical samples, and multi-omics integrative analyses remains relatively limited (Supplementary Table S1). In addition, current studies on the therapeutic effects and mechanisms of EVs are still predominantly centered on mesenchymal stem cell–derived EVs, whereas EVs derived from other cell types have been less extensively investigated, and their roles and mechanisms in HF remain insufficiently characterized. Therefore, most of the proposed mechanisms at this stage more likely reflect potential regulatory networks involving EVs, rather than definitively establishing their causal roles and contributions in cardiac pathological remodeling in HF. Future studies should integrate functional investigations of EVs across different disease stages, multi-omics approaches, and clinical evidence to further elucidate the specific roles of EVs-mediated intercellular communication in HF pathological remodeling and its translational potential.

## 4 The potential of EVs as biomarkers for HF

BNP and NT-proBNP remain the most widely used clinical biomarkers; however, their levels are influenced by multiple factors, including renal function, age, obesity, and volume status (Cao et al., 2019). High-sensitivity cardiac troponins indicate ongoing myocardial injury and serve as independent predictors of adverse outcomes in heart failure, although their interpretation in non-ischemic HF remains complex, and optimal cutoff values have not yet been standardized (Wang et al., 2020). Soluble ST2 and galectin-3 reflect myocardial fibrosis and remodeling, but their cardiac specificity is limited, and they are therefore primarily used as complementary markers for risk stratification (McDonagh et al., 2021; Heidenreich et al., 2022). Echocardiography is an essential tool for the diagnosis and prognostic assessment of HF (Senthamil Selvan et al., 2025). Cardiac magnetic resonance is regarded as the gold standard for evaluating ventricular volumes, LVEF, and myocardial fibrosis, with late gadolinium enhancement enabling the identification of myocardial scarring and risk stratification (Neilan et al., 2013). However, these imaging modalities depend on specialized equipment and operator expertise and are not well suited for bedside dynamic monitoring. These limitations highlight the need for more sensitive and dynamic molecular biomarkers. Encapsulated within EVs, miRNAs are protected from enzymatic degradation and exhibit greater stability in the circulation compared with free miRNAs, making them promising candidates for diagnostic applications. Recent advances have shown that circulating EVs-derived miRNAs play dynamic, stage-specific roles in HF pathophysiology and may serve as promising biomarkers for early diagnosis and prognosis (Zhou et al., 2020; Oh et al., 2020). In addition, although evidence for EVs-derived lncRNAs and circRNAs in HF remains limited, emerging studies suggest their potential relevance to cardiovascular diseases, including diabetic cardiomyopathy and cardiac hypertrophy, and further indicate that they may hold promise as future HF biomarkers (Singh et al., 2023). Recent studies have identified numerous circulating EVs-derived ncRNAs as noninvasive diagnostic and prognostic markers for HF (Table 1).

**TABLE 1:** Key Circulating EVs-Associated ncRNAs in the Diagnosis and Prognosis of HF.

EVs	Isolation methods/Characterization level	Clinical relevance	Clinical significance	Clinical significance	AUC/Specificity/Sensitivity/Cut-off value	Stage of development	Incremental value compared with current biomarkers	Limitations
miR-181c↑ miR-495↑ (Yang et al., 2017)	dUC + ExoRNeasy purification NTA (50–100 nm)/TEM CD9	HF caused by myxomatous mitral valve disease	EVs-derived miRNAs show higher specificity than total plasma miRNAs and are associated with progression to HF in canine MMVD	Basic research (canine model): comparison of young healthy dogs (n = 6), aged healthy dogs (n = 7), asymptomatic MMVD dogs (n = 7), and MMVD dogs with secondary CHF (n = 7), assessing differentially expressed miRNAs in EVs versus total plasma miRNAs	-	Discovery phase	Not compared	Small sample size Animal study, requiring validation in human cohorts Unclear EV origin
miR-146a↑ (Beg et al., 2017)	precipitation-based kit isolation from plasma	HF	EV-derived miR-146a more accurately reflects heart failure status than total plasma miR-146a, potentially related to HF-associated inflammatory responses	Case–control study: analysis of total plasma and EV-derived miRNAs in 40 patients with HFrEF (LVEF < 35% or NYHA class III–IV, excluding malignancies and inflammatory diseases) and 20 healthy controls	-	Discovery phase	Not compared	Small sample size Potential confounding effects from comorbidities Single time-point measurement, unable to reflect dynamic disease changes
miR-146a↑ (Halkein et al., 2013)	dUC + ExoRNeasy purification NTA (50–100 nm)/TEM CD9	HF caused by peripartum cardiomyopathy	1. Elucidates the pathogenic mechanism by which 16K PRL-induced endothelial EVs transfer miR-146a to cardiomyocytes and suppress metabolic function 2. Diagnostic value: miR-146a is specifically elevated in PPCM patients and can effectively distinguish PPCM from DCM. 3. miR-146a knockdown improves HF without affecting lactation, indicating a potential therapeutic target.	1. In vitro mechanism: HUVEC model demonstrating that 16K PRL induces endothelial miR-146a expression via the NF-κB pathway 2. In vivo validation: Stat3 knockout PPCM mouse model using antagomir/LNA to evaluate the therapeutic effects of miR-146a inhibition 3. Case–control study: Measurement of plasma miR-146a levels in PPCM (n = 38), DCM (n = 30), postpartum controls (n = 18), young healthy controls (n = 5), and PPCM patients treated with bromocriptine (n = 12)	-	Discovery phase	miR-146a was elevated exclusively in PPCM and could discriminate PPCM from DCM.	Anti-miR-146a does not fully reverse PPCM, suggesting the involvement of additional mechanisms The respective contributions of vascular injury and impaired myocardial metabolism cannot be clearly distinguished
miR-30d-5p↓ miR-126a-5p↓ (Huang et al., 2022)	dUC + filtration NTA (100 nm)/TEM	Diabetes-related HFpEF	EV-derived miR-30d-5p and miR-126a-5p are downregulated in diabetic HFpEF rats and are associated with cardiac output	Basic study: streptozotocin (STZ)-induced type 1 diabetes mellitus (T1DM) rat model. EVs from 12 HF-related circulating EVs were analyzed in control rats (n = 6) versus diabetic rats (n = 6), and their correlations with cardiac output were assessed	-	Discovery phase	Not compared	Small sample size; findings are based on an animal model and require validation in clinical patient cohorts.
miR-425↓ miR-744↓ (Wang et al., 2018)	ExoQuick precipitation-based isolation	HF cardiac fibrosis	Reduced in HF patients and associated with myocardial fibrosis, potentially through targeting TGF-β signaling	Case–control study: analysis of nine EV-derived miRNAs in plasma samples from 31 patients with acute heart failure and 31 healthy controls Cellular experiments: Ang II–treated cardiac fibroblasts were used to validate functional effects	-	Discovery phase	Not compared	Small sample size Primarily in vitro evidence, requiring in vivo validation
miR-92b-5p↑ (Wu et al., 2018a)	dUC + precipitation kit NTA (30–150 nm)/TEM CD63/Hsp70	AHF caused by DCM	miR-92b-5p is upregulated in DCM-associated acute heart failure and is negatively correlated with LVEF.	Case–control study: serum EVs were isolated from 43 patients with DCM-associated acute heart failure and 34 healthy controls. miR-92b-5p expression was quantified using qRT-PCR, and a diagnostic model was constructed to evaluate its performance	AUC 0.808 sensitivity 62.8% sensitivity 85.3% cut-off value 0.0023	Analytical validation stage	High specificity may compensate for the low specificity of BNP.	Only three miRNAs were analyzed, limiting comprehensiveness small sample size tissue-specific validation was not performed
miR-92b-5p↑ (Wu et al., 2018b)	dC + precipitation kit isolation NTA (40–150 nm)/TEM CD63/Hsp70	HFrEF	miR-92b-5p is upregulated in patients with HFrEF and is negatively correlated with LVEF.	Single-center case–control study: serum EVs were isolated from 28 patients with HFrEF hospitalized for acute heart failure (AHF) and 30 healthy controls. miR-92b-5p expression was quantified using qRT-PCR, and its diagnostic value for HFrEF as well as its correlation with LVEF were analyzed	AUC 0.844; sensitivity 71.4%; specificity 83.3% cut-off value −6.09	Analytical validation stage	Not compared	Small sample size and single-center design lack of subgroup analysis stratified by LVEF
miR-27a↑ (Xie et al., 2022)	precipitation kit-based isolation NTA (30–150 nm)/TEM CD63/Tsg101	HF survival rate	decreased in HF patients, increases after treatment, and the high-expression group has higher survival rates	Prospective cohort study: 101 patients with chronic heart failure (CHF) and 30 healthy controls were enrolled. Serum EV-derived miR-27a levels were quantified using qPCR, with a 2-year clinical follow-up for outcome assessment	AUC 0.835 sensitivity 77.23% specificity 80.00% cut-off value 1.060	Analytical validation stage	Not compared	Lack of validation for early screening value
miR-194↑ miR-34a↑ (Matsumoto et al., 2013)	dUC + filtration NTA (30–150 nm)/TEM CD63	HF incidence within 1 year after AMI	Increased at 18 days after AMI and predicts the occurrence of heart failure within 1 year	Case–control study: patients with AMI were followed after survival and classified into a heart failure (HF) group (n = 21) and a control group (n = 65) based on whether ischemic HF occurred within 1 year. Samples were collected at 18 days (median) after AMI to evaluate the predictive performance of the biomarkers	-	Analytical validation stage	Provides a post-AMI recovery window for risk stratification, which may help identify patients at high risk of heart failure and guide early follow-up	Small sample size Lack of independent validation cohort
hsa-miR-339-5p ↑ (Cheng et al., 2026)	dUC NTA (87.5 nm)/TEM	HFrEF and ventricular remodeling	Upregulated in HFrEF patients and promotes fibrosis via targeting NLRC5 and activating the PI3K/Akt pathway	1. Case–control study:** (5 HFrEF vs 5 controls) high-throughput sequencing was used to screen differentially expressed miRNAs; (40 HFrEF vs 40 controls) qPCR validation was performed, and ROC analysis was conducted to evaluate diagnostic performance 2. Cell experiments:** AC16 cardiomyocytes were used to validate the function of miR-339-5p via overexpression and inhibition	AUC 0.8749	Analytical validation stage	BNP reflects pressure overload, whereas this marker directly reflects the fibrotic process	Small sample size EV cellular origin was not clearly defined clinical utility has not been validated in large-scale clinical trials.
miR129-5p↓ (Ramachandran et al., 2017)	ExoQuick precipitation-based isolation NTA(160 nm)	HF caused by single-chamber heart disease	Negatively correlated with HF severity in single-ventricle children; independent of ventricular morphology and palliative stage	1. Cross-sectional observational study: EV-derived miR-129-5p expression was measured in 71 children aged 1 month to 7 years with single-ventricle heart disease, including a non-heart failure group (Ross ≤ 2, n = 49) and a heart failure group (Ross ≥ 3, n = 22) 2. Cell experiments: HL1 cells and hESC-derived cardiomyocytes were exposed to hypoxia or H2O2 to validate changes in miR-129-5p expression and its targeting relationship with BMPR2	AUC 0.98 sensitivity 85% specificity 100% cut-off value ≤ 0.58	Analytical validation stage	Superior to BNP/NT-proBNP, which are influenced by ventricular morphology, and therefore has a broader applicability	Ross score has not been specifically validated in single-ventricle populations single-center study
miR-27a-5p↑ miR-139-3p↓ (Chen et al., 2025)	no clear EV isolation method described	CHF with Hyperuricemia	Differential expression in CHF patients with hyperuricemia; combined markers outperform single-marker detection	Single-center case–control study: high-throughput sequencing was performed to identify differentially expressed miRNAs between CHF-HUA patients (n = 6) and healthy controls (n = 6). An independent cohort (CHF-HUA, n = 24; healthy controls, n = 24) was used for qRT-PCR validation of candidate miRNAs, and a dual-miRNA combined diagnostic model was constructed	AUC 0.899 sensitivity 79.2% specificity 91.7% cut-off value 0.000	Analytical validation stage	Not compared	Single-center, small-sample-size study Restricted to the Chinese Han population
miR-144-3p↓ (Wang C. K. et al., 2024)	exoRNeasy kit-based isolation NTA (45–150 nm)/TEM CD63/TSG101	HF patients with depressive symptoms	Decreased in HF patients and negatively correlated with the severity of depressive symptoms	Single-center case–control study 1. Differential miRNA profiles were identified between HF patients with depressive symptoms (HF-DS, n = 6) and HF patients without depressive symptoms (HF-NDS, n = 6) 2. An independent cohort (HF-DS, n = 20; HF-NDS, n = 20) was used for qRT-PCR validation, and ROC curve analysis was performed to evaluate diagnostic performance	AUC 0.763 sensitivity 90.0% specificity 50.0% cut-off value 0.206	Analytical validation stage	Not compared	Low specificity (50%) high false-positive rate needs combination
has_circ_0097435 (Han et al., 2020)	Hieff™ rapid exosome isolation kit-based isolation	HF	Upregulated in EVs from heart failure patients and promotes cardiomyocyte apoptosis	Below is the SCI-style English translation, keeping structure and academic rigor 1. Observational case–control study:** peripheral blood high-throughput sequencing was performed to screen differentially expressed circRNAs between HF patients (n = 5) and controls (n = 4). qPCR validation was conducted in an expanded cohort (HF, n = 40; controls, n = 40). In addition, plasma EV levels were measured in an independent subgroup (HF, n = 15; controls, n = 15) 2. In vitro study:** in doxorubicin (DOX)-induced AC16 cardiomyocyte models, gain- and loss-of-function experiments of the circRNA were performed. RNA pull-down and AGO2 immunoprecipitation assays confirmed a ceRNA mechanism by which the circRNA promotes cardiomyocyte apoptosis	-	Analytical validation stage	Not compared	Validation was limited to in vitro cellular models, lacking in vivo functional evidence from cardiomyocyte-specific knockout mice small sample size limited its power for translation into a clinical predictive model the tissue origin of circulating EVs remains unclear
miR-223-3p/miR-378c (Limpitikul et al., 2025)	dUC CD81/ALIX/AGO2/TGFβi	HHF or CVD within 1 year after ACS	An independent predictor of HHF or CVD within 1 year in patients with ACS.	1. Prospective cohort study (MERLIN-TIMI 36, n = 4541):** with a median follow-up of 12 months, 313 individuals reached the primary endpoint. Cox regression analysis was performed to assess the association between 21 candidate miRNAs and HHF/CVD. 2.Independent validation cohort (MGH, n = 27):** small extracellular vesicles (sEVs) were isolated using differential ultracentrifugation	-	Clinical validation phase	After adjustment for NT-proBNP, miR-223-3p and miR-378c remained significantly associated with HHF/CVD, suggesting that they provide incremental predictive value independent of BNP.	Limited generalizability of the MERLIN trial population lack of serial echocardiographic data to assess changes in ventricular remodeling; incomplete baseline LVEF data small validation cohort with limited long-term outcome data

Most current studies remain at the discovery stage, primarily using high-throughput sequencing or microarray technologies to screen differentially expressed EV-derived ncRNAs in animal models and patients with HF. However, these studies commonly suffer from small sample sizes, lack of independent validation cohorts, and non-uniform detection methods. In a canine MMVD model, miR-181c and miR-495 in EVs were associated with HF progression, and EVs-derived miRNAs showed higher specificity than those in total plasma (remaining significant at an FDR of 5%, whereas all signals disappeared at an FDR of 15% in total plasma), suggesting a lower false-positive rate for EV-derived miRNAs as biomarkers (Yang et al., 2017). Beg et al. found that miR-146a was elevated in EVs from patients with HF, whereas no change was observed in total plasma (p = 0.05), further supporting that EV-derived miRNAs better reflect HF status; however, the study was limited by a small sample size and a borderline p value (Beg et al., 2017). Halkein et al. demonstrated that miR-146a was specifically upregulated in patients with peripartum cardiomyopathy (PPCM) and could effectively distinguish PPCM from dilated cardiomyopathy, while animal experiments showed that miR-146a knockdown improved cardiac function without impairing lactation (Halkein et al., 2013). In a diabetic HFpEF rat model, miR-30d-5p and miR-126a-5p in EVs were downregulated and correlated with cardiac output, although further clinical validation is required (Huang et al., 2022). In addition, miR-425 and miR-744 in EVs from patients with HF were reduced and were reported to regulate myocardial fibrosis via targeting TGF-β1. However, these findings were based on small sample sizes and primarily *in vitro* evidence (Wang et al., 2018). Overall, biomarkers at this stage still lack independent external validation, and clinical translational evidence remains limited.

A small number of studies have entered the validation stage and have reported preliminary diagnostic performance. miR-92b-5p has been validated in two independent cohorts of DCM-related acute HF and HFrEF, and its relatively high specificity is considered to complement the limitations of BNP (Wu et al., 2018a; Wu et al., 2018b). MiR-27a is downregulated in patients with HF, upregulated after treatment, and high expression is associated with a higher 2-year survival rate, suggesting its potential value in both diagnosis and prognostic assessment; however, these findings are based on a single-center cohort of 101 patients and still require multicenter validation (Xie et al., 2022). MiR-194 and miR-34a are elevated 18 days after acute myocardial infarction and can predict HF onset within 1 year, providing potential value for risk stratification. However, the sample size is small and external validation is lacking (Matsumoto et al., 2013). Hsa-miR-339-5p in HFrEF promotes fibrosis by targeting NLRC5 and activating the PI3K/Akt pathway, unlike BNP, which reflects pressure overload, this marker directly reflects fibrotic progression, but it is also limited by a single-center small cohort (Cheng et al., 2026). MiR-129-5p shows excellent diagnostic performance in children with single-ventricle heart disease and is not affected by ventricular morphology, outperforming BNP (Ramachandran et al., 2017). The combined detection of miR-27a-5p and miR-139-3p shows good diagnostic accuracy in CHF patients with hyperuricemia (Chen et al., 2025). MiR-144-3p has been shown to be associated with more severe depressive status and can identify HF patients with comorbid depression. However, its specificity is only 50%, with a high false-positive rate, suggesting that it should not be used alone and should be combined with other biomarkers (Wang R. et al., 2024). In addition, EV-derived circRNA hsa_circ_0097435 is significantly upregulated in patients with HF and promotes cardiomyocyte apoptosis via a miRNA sponge mechanism, however, current evidence is mainly based on *in vitro* experiments and small clinical cohorts, and its *in vivo* function remains unclear (Han et al., 2020).

Only a limited number of studies have entered the clinical validation stage. In the MERLIN-TIMI 36 prospective cohort study (n = 4541), EV-derived miR-223-3p (decreased) and miR-378c (increased) remained significantly associated with 1-year HF hospitalization or cardiovascular death in patients with acute coronary syndrome (ACS) after adjustment for NT-proBNP, hs-TnT, and hs-CRP, and these findings were validated in an independent cohort, suggesting incremental prognostic value beyond traditional biomarkers (Limpitikul et al., 2025). However, it should be noted that the study population consisted of patients with ACS rather than those with previously diagnosed HF, and no EV-derived ncRNA biomarker has yet been implemented in clinical practice for HF.

Compared with miRNAs, EV-derived lncRNAs and circRNAs face greater challenges in clinical translation, including lower abundance in circulation, higher requirements for detection sensitivity, and difficulties in primer design due to high sequence homology between circRNAs and linear RNAs. In addition, circRNA detection often relies on complex experimental procedures such as RNase R treatment. Furthermore, the lack of standardized internal controls, variability in RNA extraction efficiency, and confounding factors such as hemolysis and repeated freeze–thaw cycles further reduce reproducibility. Collectively, these issues limit their clinical application.

In summary, EV-derived ncRNAs show considerable potential for the diagnosis and prognostic evaluation of heart failure. However, current evidence remains largely at the discovery and analytical validation stages. Major limitations include single-center small-sample studies, lack of independent external validation, insufficient direct comparison with conventional biomarkers, and the absence of standardized detection protocols. Future studies should focus on establishing standardized workflows (including sample collection, EV isolation, RNA extraction, and internal reference selection) and validating their incremental value over existing biomarkers through large-scale, multicenter, and prospective clinical studies.

## 5 Therapeutic potential of EVs in HF

Despite advances in HF treatment, mortality remains high. EVs have attracted increasing attention in cardiovascular research due to their biocompatibility. Preclinical studies have suggested potential cardioprotective effects of EVs in experimental HF models, particularly those derived from stem cells or engineered delivery systems. However, evidence from cardiovascular clinical trials remains limited, and EVs-based therapies for HF are still in the early stages of clinical translation (Table 2).

**TABLE 2:** Summary of preclinical studies on exosome-based HF therapy.

EVs	Isolation methods/Characterization level	Cell source	Target gene	Model	Cardiac functional endpoints/Cardiac effect
miR-182 (Zhao et al., 2019)	dUC NTA(50–150 nm)/TEM CD9,/CD63/TSG101/Alix	BMSC	TLR4	I/R mouse model	LVEF↑/LVFS↑/infarct size↓ Modulating macrophage polarization Inflammatory levels↓
miR-125a-5p (Gong S. et al., 2024)	dUC NTA(120–150 nm)/TEM Alix/Tsg101		TRAF6/IRF5	AMI rat model	LVEF↑18%/LVFS↑/10%/infarct size↓24% Macrophage m2 polarization↑ Cardiac repair after infarction↑
miR-139–3p (Ning et al., 2023)	dUC TEM(100–200 nm) CD63/CD81/TSG101/Alix		STAT1	AMI rat model	LVEF↑24%/LVFS↑20%/infarct size↓20% Modulating macrophage polarization Cardiac repair after infarction↑ Cardiac function↑ Infarct area↓
miR-129-5p (Yan et al., 2022)	dUC TEM(30–140 nm) CD81/TSG101		TRAF3/NF-κB	HF mouse model HL-1 (OGD)	LVEF↑/LVFS↑/infarct size↓ Apoptosis↓ Oxidative stress↓
miR-132 (Ma T. et al., 2018)	precipitation (Total Exosome Isolation Reagent) TEM(<150 nm) CD63/CD9		RASA1	AMI mouse model	LVEF↑20%/LVFS↑10% Ecs angiogenesis↑ Neovascularization around the infarct↑
miR-25–3p (Du et al., 2024)	dUC NTA(130–150 nm)/TEM CD63/CD9		JAK2/STAT3	MIRI rat model	infarct size↓ Modulating macrophage polarization Inflammation↓
miR-29c (Li H. et al., 2020)	dUC CD9/CD63/Alix		PTEN/Akt/mTOR	I/R mouse model NRCM(H/R)	Infarct size↓ Regulating autophagy changes
miR-30e (Pu et al., 2021)	dUC NTA (44–107 nm)/TEM CD9/CD63/CD81/Alix		LOX1/NF-κB p65/Caspase-9	HF rat model	LVEF↑/LVFS↑/infarct size↓ Myocardial cell apoptosis↓ Fibrosis↓
lncRNA GAS5 (Ren and Zhao, 2024)	UC NanoFCM (36–140 nm)/TEM CD63/CD81		UL3/Hippo	HF rat model H9C2(hypoxic)	LVEF↑/LVFS/Oxidative stress↓ Apoptosis↓ Fibrosis↓ Iron deposition↓
miR-21a (Wei et al., 2021)	UC NTA/TEM CD9/CD63/ALIX/TSG101		PTEN	I/R mouse model H9c2 (A/R)	LVEF↑/LVFS↑, infarct size↓ Cell death↓ Inflammation↓
mir-214-3p (Zhu et al., 2023)	dUC + precipitation NTA(30–150 nm)/TEM CD63/CD81/TSG101/Calnexin	HUCMSCs	PTEN/p-AKT	AMI rat model	LVEF↑/LVFS↑ Myocardial cell apoptosis↓ Infarct area↓ Cardiac function↑ Myocardial cell survival↑ Ecs functions↑
miR-29b (Yuan et al., 2023)	dUC NTA/DLS (30–90 nm)/TEM CD63/CD81/TSG101/Calnexin		-	MI mouse model	LVEF↑/LVFS↑ Inflammation↓ Infarct area↓ Fibrosis↓ Cardiac function↑
miR-24-3p (Zhu F. et al., 2022)	dUC NTA/TEM CD63/TSG101		Plcb3/NF-κB	AMI mouse model RAW264.7 (LPS)	LVEF↑/LVFS↑, infarct size↓ M2 macrophage polarization↑
miR-1246 (Wang Z. et al. 2021)	dUC NTA (30–150 nm)/TEM CD63/TSG101/ALIX/Calnexin		PRSS23	CHF rat model NRCM/HUVEC(OGD)	LVEF↑/LVFS↑ Cardiac function↑ Reduction in myocardial infarction area↓ Apoptosis↓ Angiogenesis↑
EGR1 siRNA (Huang et al., 2025)	dUC DLS/ELS/TEM CD63/HSP70/Calnexin		-	MI/RI mouse model CMs (OGD)	LVEF↑/LVFS↑ Myocardial cell viability↑ Levels of oxidative stress↓ Regulating mitochondrial autophagy
miR-*221* miR-*222* (Lee et al., 2025)	dC + ExoQuick-TC precipitation NTA(122 nm)/TEM CD9/CD63	ADSC	BNIP3-MAP1LC3B-BBC3/PUMA	I/R mouse model H9C2(H/R)	LVEF↑/LVFS↑/infarct size↓ Regulating mitochondrial autophagyregulate apoptosis
miR-205 (Wang T. et al., 2023)	dC + ExoQuick-TC precipitation NTA(100 nm)/TEM CD63/CD9/CD81/TSG101/Alix		HIF-1α′VEGF	MI mouse model HMEC-1(OGD)	LVEF↑/LVFS↑ Fibrosis↓ Angiogenesis↑
miR-93–5p (Liu et al., 2018)	dC + ExoQuick-TC precipitation NTA (100 nm)/TEM CD9/CD63/TSG101		Atg7′TLR4	AMI rat model H9C2(hypoxic model)	infarct size↓ Autophagy↓ Inflammatory cytokines↓
miR-182/183 (Yang et al., 2023)	dUC + sucrose gradient + filtration NTA(50–150 nm)/TEM CD9/CD63	Cortical bone stem cells	RASA1	MI mouse model BMDM(LPS)	LVEF↑/LVFS↑ Macrophage-mediated Repair polarization Metabolic reprogramming Myocardial repair↑
miR-210 (Ma X. et al., 2018)	dUC + filtration NTA(50–150 nm) CD63	EPC	-	EC(H/R)	Mitochondrial function↑
miR-181b (de Couto et al., 2017)	ultrafiltration + PEG precipitation + filtration NTA(150 nm)/DLS/TEM/CD63/Alix/HSP70	CDC	PKCδ	MI rat/pig models Macrophages	infarct size↓ Modulate the polarization state of macrophages Infarct area↓
miR-181b-5p (Li et al., 2023)	EVs: dUC + filtration TEM(75–82 nm)/NanoFCM CD9/CD63/Alix/Calnexin	Macrophage	CCR2 macrophage subpopulations	I/R rat/pig/mouse models Co-culture of BMDMs and HCAECs	LVEF↑/LVFS↑/infarct size↓ Angiogenesis↑ Ecs migration↑ Regulate myocardial repair
miR-222 (Jansen et al., 2015)	dC	ECs	ICAM-1	ApoE−/− mouse model HCAEC (TNF-a,oxLDL) Plasma validation of miR-222 in patients without CAD and with stable CAD	Vascular inflammation↓
miR-664a-3p (Wang et al., 2025)	dUC + filtration NTA(50–200 nm)/TEM CD63/CD81/TSG101	Healthy young adult plasma	TGF-β/SMAD4	MI mouse model CFs(TGF-β1)	LVEF↑/LVFS↑ Protecting the heart from fibrosis Improve cardiac function
miR-100 (Li et al., 2024)	dUC + filtration TFF/TEM/Zetasizer	Pulmonary spheroid cells	Cd36	AMI mouse/pig models	LVEF↑/LVFS↑ CD36↓ Compensatory increase in cardiac glucose utilization ATP production↑ Cardiac contractility↑

### 5.1 Stem cell-derived EVs as potential therapeutic candidates

Stem cell therapies have shown promise; however, their clinical translation is limited by low engraftment efficiency and potential tumorigenic risks. Recent reviews have indicated that EVs derived from MSCs, cardiac progenitor cells, and iPSCs retain key reparative properties of their parent cells while avoiding risks associated with cell transplantation, making them promising strategies for cardiac regeneration (Yazbeck et al., 2025). Numerous preclinical studies suggest that stem cell-derived EVs may mediate many of the beneficial paracrine effects of transplanted stem cells and have shown cardioprotective properties in experimental HF and myocardial injury models (Yahyazadeh et al., 2024). Several EVs-derived miRNAs play crucial roles in HF, including miR-182 (Zhao et al., 2019), miR-125a-5p (Gong Z. T. et al., 2024), miR-24-3p (Zhu F. et al., 2022), miR-25-3p (Du et al., 2024), miR-139-3p (Ning et al., 2023), miR-182/183 (Yang et al., 2023), and miR-196a-5p, miR-425-5p (de Almeida Oliveira et al., 2022) induce macrophage polarization, reduce inflammation, improve structural remodeling, and confer cardioprotective effects. MiR-210 (Ma X. et al., 2018), miR221/222 (Lee et al., 2025)*,* miR-29c (Li T. et al., 2020) regulate mitochondrial dysfunction and cardioprotection by reducing mitochondrial fragmentation and apoptosis while maintaining mitochondrial autophagy balance. MiR-129-5p (Yan et al., 2022), miR-30e (Pu et al., 2021), and lncRNA GAS5 (Ren and Zhao, 2024) suppress oxidative stress and apoptosis, protecting the heart from failure. MiR-29b (Yuan et al., 2023), miR-205 (Wang T. et al., 2023), and miR-1246 (Wang Z. et al., 2021) suppress myocardial fibrosis and promote angiogenesis, facilitating cardiomyocyte repair and regeneration to enhance cardiac function. These findings underscore the therapeutic potential of stem cell-derived EVs in HF, particularly through miRNA-mediated mechanisms.

Future efforts should focus on optimizing EVs composition to enhance therapeutic efficacy and enable more personalized treatment strategies. Beyond stem cell-derived EVs, EVs from other cell types also exhibit therapeutic potential in HF, including macrophage-derived miR-181b-5p (Li et al., 2023), CDC-derived miR-181b (de Couto et al., 2017), ECs-derived miR-222 (Zhang et al., 2020, Jansen et al., 2015), and miR-664a-3p from young healthy human plasma (Wang et al., 2025). In addition, EVs may serve as supportive tools for regenerative therapies. Human cardiac fibroblast-derived EVs have been shown to promote the maturation of hiPSC-derived cardiomyocytes through AMPK-dependent metabolic reprogramming, thereby enhancing their therapeutic efficacy after myocardial infarction (Qin K. et al., 2026).

Recently, Capricor Therapeutics announced positive results from its phase III clinical trial HOPE-3 (NCT05126758) of Deramiocel (CAP-1002) for the treatment of Duchenne muscular dystrophy (DMD). Deramiocel is an allogeneic cell therapy derived from CDCs that exerts immunomodulatory, anti-inflammatory, and anti-fibrotic effects through EV secretion. Its bioactive miRNAs are reported to regulate macrophage phenotypes and promote tissue repair. HOPE-3 trial showed that this therapy may slow cardiac functional decline and reduce myocardial fibrosis, suggesting potential relevance to HF-related pathological processes. However, its indication is DMD-associated cardiomyopathy, which differs from HF in etiology and disease progression, and its efficacy cannot be directly extrapolated to HF populations. The BLA application, CRL feedback, and phase III validation process of CAP-1002 provide valuable experience for EV-based clinical development; however, HF still requires an independent evidence framework for efficacy and safety. Importantly, despite encouraging findings in DMD-associated cardiomyopathy, clinical evidence supporting EV-based therapies in HF remains scarce, and their efficacy and safety in HF populations have yet to be established.

### 5.2 EVs-mediated drug delivery strategies

EVs mediate intercellular communication and have garnered increasing interest as drug delivery vehicles (Liang et al., 2025). Several approaches have been developed to load therapeutic agents into EVs, which are primarily categorized into two types. Passive preloading involves introducing synthetic constructs such as miRNA-expressing plasmids into EV-producing cells or co-incubating therapeutic agents with source cells. While this method preserves vesicle integrity, drug loading efficiency is generally low (Pascucci et al., 2014; Jurgielewicz et al., 2021). For instance, EVs derived from MSCs overexpressing miR-214 target PTEN and activate p-AKT signaling to promote cardiac repair in AMI (Zhu et al., 2023). Similarly, ADSC-derived EVs enriched with miR-93-5p prevent cardiac injury by suppressing autophagy and inflammation (Liu et al., 2018). Building on these strategies, researchers have developed molecular recognition systems for active mRNA loading. Luo et al. utilized the L7Ae–box C/D RNA interaction system to actively recruit Ndufs1 and VegfA mRNAs into EVs during biogenesis, increasing loading efficiency several thousand-fold while maintaining EV integrity. In a mouse MI model, these engineered EVs improved mitochondrial function, promoted angiogenesis, and enhanced cardiac function (Luo et al., 2026). Direct loading of therapeutic agents into purified EVs has become a more widely used strategy (Ingato et al., 2016). Technologies such as electroporation, cell nanopores, ultrasonication, and chemical transfection are commonly applied to load miRNAs, siRNAs, or drugs into EVs (Gu et al., 2024; Yang et al., 2020). For example, electroporation-loaded miR-132 into MSC-derived EVs enhances endothelial angiogenic behavior *in vitro* and preserves cardiac function in a mouse MI model (Ma T. et al., 2018). EV targeting efficiency can be further improved by covalently conjugating targeting ligands or engineering EV membranes with specific peptides (Zeng et al., 2023). Vandergriff et al. demonstrated that EVs targeted to the infarcted heart via DOPE–NHS linkers conjugated with cardiac homing peptides significantly reduce fibrosis and infarct size while promoting cell proliferation and angiogenesis (Vandergriff et al., 2018). Mentkowski et al. engineered CDCs to express cardiomyocyte-specific peptides on isolated EV surfaces, enhancing cardiomyocyte uptake, reducing apoptosis, and improving cardiac retention (Mentkowski and Lang, 2019). However, rapid clearance by the liver and spleen limits systemic delivery efficiency. To address this, a two-step EV delivery strategy has been proposed, involving surface modification of EVs to evade macrophage phagocytosis. For instance, EVs engineered with CD47 and loaded with miR-21a exhibit improved internalization into cardiomyocytes, suppress inflammation, and enhance cardiac function (Wei et al., 2021).

EVs are commonly administered via systemic or local injection routes (Gallet et al., 2017). Encapsulation of EVs within functional hydrogels can prolong their retention. Hydrogels such as shear-thinning gels and PGN hydrogels enable sustained EV release in the myocardial ischemic border zone for more than 21 days, resulting in more pronounced improvements in cardiac function and greater reductions in fibrosis, cell death, and inflammation compared with EVs alone (Chen et al., 2018; Han et al., 2019). EVs-loaded cardiac patches have also been developed for the repair of MI-induced injury. Encapsulation of anti-fibrotic miR-29b mimics into gelatin-based biocompatible microneedle (MN) patches increases EV retention, reduces infarct size, alleviates inflammation, and suppresses fibrosis (Yuan et al., 2023). Similarly, human umbilical cord MSC-derived MN patches encapsulating EGR1 siRNA demonstrate cardioprotective effects in MI/RI models by enhancing cardiomyocyte viability, alleviating oxidative stress, and regulating mitochondrial autophagy (Huang et al., 2025). However, EVs delivery via myocardial injection or cardiac patches typically requires invasive procedures, limiting clinical applicability. Thus, minimally invasive or non-invasive delivery strategies are receiving increasing attention. A minimally invasive EV spray, based on MSC-derived EVs and biomaterials, improves cardiac function, reduces fibrosis, and promotes endogenous vascular regeneration in an AMI mouse model (Yao et al., 2021). Recently, stem cell-derived EVs nebulization therapy (SCENT) significantly reduced fibrotic tissue and preserved left ventricular wall thickness in mouse and pig AMI models. Notably, inhaled EVs began accumulating in the heart within 60 min, with substantial retention even after 48 h under repeated administration. This non-invasive and repeatable approach provides a foundation for future clinical translation (Li et al., 2024). Although these delivery strategies show promising preclinical potential, most remain at an early stage of translational development.

## 6 Pharmacological implications: integrating EVs into current HF treatment paradigms

### 6.1 Bidirectional interactions between HF drugs and EVs

Current evidence indicates a close relationship between core pharmacological therapies for heart failure and EVs. HF drugs can influence EV biogenesis, cargo composition, and functional activity, while EVs, as natural delivery vehicles, are also being explored to enhance targeted drug delivery efficiency. Specifically, sacubitril/valsartan increases EV production in cardiomyocytes and downregulates miR-181a in EVs (Vaskova et al., 2020). Empagliflozin preconditioning of MSCs enhances EVs secretion, and the resulting EVs exhibit stronger therapeutic effects compared with those from untreated cells (Jing et al., 2025). Similarly, dapagliflozin can alter EV composition by regulating lncRNA/miRNA expression profiles (Huang et al., 2026). Carvedilol has also been shown to enhance EV biogenesis and functional activity in macrophages (Chen et al., 2019). On the other hand, β-adrenergic receptor blockers (atenolol and metoprolol) have been used for EV functional modification to improve their targeting efficiency toward cardiomyocytes (Park K. C. et al., 2025). Notably, telmisartan not only acts on the classical RAS pathway but also modulates EV-mediated pathological effects. In spontaneously hypertensive rats, circulating EVs induce cardiomyocyte hypertrophy, whereas telmisartan significantly suppresses this process, suggesting that RAS inhibitors may exert cardioprotective effects in part through regulation of EV-associated signaling (Yu et al., 2023). Overall, current HF drugs can modulate EV biogenesis, composition, and function, while EVs also hold promise as drug delivery platforms to improve therapeutic efficacy. Therefore, when developing EV-based therapeutic strategies and conducting clinical trials, potential drug–EV interactions should be carefully considered due to their possible impact on efficacy and safety.

### 6.2 EVs-regulated signaling pathways and their associations with established HF drug targets

ACEI/ARB, MRA, SGLT2 inhibitors, ARNI, and β-blockers exert cardioprotective effects in part through specific signaling pathways, such as NF-κB, TGF-β, p38 MAPK, and PI3K/Akt, which are also key nodes involved in EV-mediated myocardial repair and remodeling, suggesting a mechanistic convergence at the pharmacological level. ACE inhibitors (benazepril) attenuate ventricular remodeling by inhibiting NF-κB and TGF-β signaling (Yan et al., 2013), whereas ARBs (olmesartan and telmisartan) act through suppression of the TAK1/p38 MAPK pathway (Wu et al., 2016). Correspondingly, EVs derived from cardiosphere-derived cells carrying circRNA_000203 can upregulate Gata4 by sponging miR-26b-5p and miR-140-3p, thereby modulating NF-κB signaling and promoting cardiomyocyte hypertrophy (Li H. et al., 2020). These findings indicate that drugs and EVs act on overlapping signaling networks, providing mutual mechanistic references in cardiac remodeling. Similarly, spironolactone exerts anti-fibrotic effects by inhibiting the TGF-β1/Smad2/3 signaling pathway (Luo et al., 2011; Wang et al., 2022). EVs also exhibit bidirectional regulation of this pathway. Specifically, cardiomyocyte-derived EVs aggravate fibrosis via miR-494-3p–mediated activation of this pathway (Tang et al., 2023), whereas EVs derived from plasma of healthy adults carry miR-664a-3p, which suppresses SMAD4 and exerts anti-fibrotic effects (Wang et al., 2025). This shared pathway involvement suggests that EVs may functionally parallel MRA-mediated anti-fibrotic mechanisms. In addition, SGLT2 inhibitors activate AMPK and inhibit NF-κB signaling, thereby improving mitochondrial function and reducing inflammation (Gong S. et al., 2024; Quagliariello et al., 2021; Yan et al., 2026). ARNI and β-blockers reduce apoptosis through activation of the PI3K/Akt pathway (Jin et al., 2024; Wang Q. et al., 2021; Baraka et al., 2021). Consistently, BMSC-derived EVs improve cardiomyocyte apoptosis and fibrosis via miR-30e–mediated LOX1 suppression and miR-29c–regulated PTEN/Akt/mTOR signaling (Li T. et al., 2020; Pu et al., 2021). Collectively, these examples further demonstrate that EVs and established HF therapies share key signaling pathways, suggesting that EVs are not independent mechanisms but rather potential modulators and complements of cardioprotective pharmacological processes.

### 6.3 EVs as pharmacodynamic biomarkers

In addition to their roles in diagnosis and prognostic assessment, dynamic changes in EVs-derived ncRNAs may reflect biological responses to pharmacological interventions, highlighting their potential as pharmacodynamic biomarkers. Existing studies have shown that after sacubitril/valsartan treatment, miR-181a levels in circulating EVs are reduced and are associated with improvements in cardiac function, suggesting that EV-derived ncRNAs may be useful for monitoring therapeutic responses (Vaskova et al., 2020). In addition, Wenyang Zhenshuai Granules promote EV secretion from cardiomyocytes and increase miR-155 content, thereby inhibiting the p38 MAPK pathway and reducing cardiomyocyte apoptosis. Enalapril has also been reported to exhibit similar cardioprotective effects in the same study (Peng et al., 2025). These findings indicate that drug-induced alterations in EV composition not only contribute to therapeutic effects but may also reflect the biological response of the host to treatment. Although direct evidence remains limited, EV-derived ncRNAs can reflect intercellular communication and molecular remodeling processes in the myocardium. Based on these properties, EV-derived signals may serve as important complements to existing biomarkers for monitoring treatment response, risk stratification, and future optimization of personalized therapeutic strategies. However, their clinical utility still requires further validation in large-scale prospective studies.

### 6.4 Positioning of EVs-based therapies relative to conventional and emerging HF pharmacotherapies

EVs-based therapies differ markedly from conventional HF drugs in terms of administration routes and pharmacokinetic characteristics, with their tissue distribution and therapeutic efficacy being highly dependent on the delivery route. For instance, following intravenous injection, less than 5% of small EVs remain in circulation within 5 min, with a blood half-life of approximately 2 h (Su et al., 2025). In contrast, after intrapericardial administration, EVs can penetrate the epicardium within 2 h and migrate into the myocardium, with retention in the cardiac region lasting at least 7 days (Zhu et al., 2021). At present, dosing in preclinical studies varies substantially (10^8^–10^10^ particles per mouse), and a standardized dose conversion framework is still lacking (Gupta et al., 2021). In terms of clinical translation, the SECRET-HF trial (NCT05774509) has adopted an intravenous dosing regimen of 20 × 10^9^ particles/kg every 3 weeks for a total of 3 administrations, with preliminary results indicating safety and feasibility, thereby providing a reference for future dose optimization (Menasché et al., 2024).

Combination strategies involving EVs and existing HF therapies also show considerable potential. In a mouse LAD infarction model, both metoprolol and empagliflozin combined with mitochondria-enriched extracellular vesicles further improved cardiac function compared with monotherapy, suggesting potential additive effects (Abdi et al., 2025). In addition, exercise training combined with MSC-derived EVs further enhances cardiac function and structural recovery after myocardial ischemia (ShamsEldeen et al., 2025). Although related studies remain limited, evidence from other disease contexts also supports the feasibility of such combination strategies. In a hypertensive chronic kidney disease mouse model, combination therapy with RLX (a recombinant human relaxin with anti-fibrotic properties) and BMSC-derived EVs produced stronger anti-fibrotic effects compared with BMSC-EVs alone (Li et al., 2021). Overall, EV-based therapies are not intended to replace existing HF drugs but are more likely to serve as adjunctive strategies on top of standard therapy. Unlike conventional drugs that primarily target neurohormonal activation and hemodynamic abnormalities, EV-based therapies focus more on promoting myocardial repair, suppressing inflammation and apoptosis, and enhancing angiogenesis, thereby holding potential for application in patients requiring tissue repair and regeneration.

## 7 Challenges and outlook

### 7.1 Technical challenges: isolation, purification, and standardization

Current EV purification techniques, including differential centrifugation, density gradient centrifugation, size exclusion chromatography, ultrafiltration, and immunoprecipitation, are time-consuming, costly, and yield low purity (Laura Francés et al., 2023; Alvi et al., 2021). EVs are highly heterogeneous, and existing isolation methods primarily rely on particle size, density, and discrete surface markers, making it impossible to distinguish between exosomes, microvesicles, exosomal subpopulations, and other bioactive components (Tang et al., 2017). This limitation hinders biological characterization and therapeutic standardization. Quantification methods such as resistive pulse sensing, tunable resistive pulse sensing, dynamic light scattering, and nanoparticle tracking analysis lack standardization, thereby limiting the robustness and comparability of measurements (Zhao and Huang, 2024). Overcoming these technological obstacles will require the development of novel methods for efficient EV isolation and purification that enable accurate identification and characterization of distinct EV subpopulations.

### 7.2 Strategies for EVs delivery to the heart and associated challenges

EVs delivery routes directly influence their cardiac distribution and therapeutic efficacy. Intravenous injection is simple and widely used, but EVs have a short systemic half-life and are rapidly cleared by macrophages in the liver, spleen, and lungs, resulting in limited cardiac accumulation. Intrapericardial injection enables efficient local delivery, allowing EVs to penetrate the epicardium within a short time and persist in the myocardium for extended periods, but its invasiveness limits clinical applicability. Inhalation delivery offers a non-invasive alternative, enabling EVs to cross the alveolar–capillary barrier and enter systemic circulation, with evidence of improved cardiac function in MI models, although delivery efficiency remains suboptimal. Oral administration is significantly affected by the gastrointestinal environment and shows low cardiac targeting efficiency, and is therefore mainly explored for gastrointestinal diseases. Intranasal delivery has the potential to bypass the blood–brain barrier, but its application in cardiac delivery remains insufficiently validated (Li et al., 2024; Su et al., 2025; Zhu et al., 2021; Ayanji et al., 2026). Overall, different administration routes involve a trade-off between delivery efficiency and clinical feasibility. Invasive approaches achieve higher local delivery efficiency but are limited in translational potential, whereas non-invasive strategies are more clinically attractive but generally suffer from inadequate targeting specificity and *in vivo* stability. In addition, most current EVs delivery studies remain at the preclinical stage and lack systematic pharmacokinetic and dose–response data, which limits robust evaluation of their clinical translation.

### 7.3 Safety considerations and comparison of delivery systems

EVs-based therapies have demonstrated preliminary safety across multiple disease areas in clinical studies, however, their cardiovascular application still faces several challenges. Allogeneic EVs may trigger host immune responses, including complement activation and cytokine release, and their immunogenicity is closely associated with cellular origin and surface properties (Lee et al., 2024). In addition, MSC-derived EVs can express tissue factor and exhibit potential procoagulant activity, and high-dose intravenous administration has been reported to induce pulmonary embolism in animal studies (Wright et al., 2023). Long-term safety remains unclear, with potential risks including off-target effects, tumor promotion, and pro-fibrotic responses. Several adverse events have also been observed in clinical studies. EVs derived from dendritic cells have been associated with three cases of delayed hypersensitivity reactions, five cases of grade 1 fever and injection-site inflammation, and one case of grade 3 hepatotoxicity in clinical trials (Morse et al., 2005; Escudier et al., 2005; Besse et al., 2016). In addition, studies in non-cardiovascular applications have reported severe adverse events, including local necrosis and vasculitis (Queen and Avram, 2025).

In terms of safety comparisons among delivery systems, EVs exhibit lower immunogenicity, good biocompatibility, and no risk of genomic integration compared with viral vectors and lipid nanoparticles (Chen et al., 2021; Tenchov et al., 2022). However, their clinical translation is still limited by complex manufacturing processes, insufficient batch-to-batch consistency, and challenges in large-scale production. Viral vectors provide high delivery efficiency but are associated with strong immunogenicity. Lipid nanoparticles have been widely used clinically but are limited by hepatic accumulation, poor tissue targeting, and restricted bioavailability (Eygeris et al., 2022). Overall, different delivery systems show clear trade-offs between safety and manufacturability, and no single platform currently achieves both high safety and high efficiency (Ma et al., 2026). Although part of the evidence is derived from non-cardiac studies, it still provides useful reference for safety evaluation and cardiovascular translation of EV-based therapies.

### 7.4 Clinical integration and application scenarios

EVs-based biomarkers show cross-stage application potential in heart failure management. For early diagnosis, the miRNA profile in plasma EVs from patients with HFrEF is significantly altered, and differential expression of multiple miRNAs, such as hsa-miR-22-5p, may serve as potential screening biomarkers (Cheng et al., 2026). With advances in single-vesicle analysis technologies, specific EVs subpopulations have also demonstrated diagnostic value. For example, DSCAML1-positive EVs are significantly enriched in patients with AMI, suggesting dual potential as both biomarkers and therapeutic targets (Liu et al., 2026). For disease progression and risk stratification, changes in EVs abundance and cellular origin are associated with worsening cardiac function. For instance, elevated levels of leukocyte-derived EVs in patients with HFmrEF are significantly associated with an increased risk of further decline in systolic function (Gąsecka et al., 2025). In prognostic prediction, exosomal miRNAs within a specific time window after AMI, such as miR-194 and miR-34a, are closely associated with long-term HF development and may support early risk assessment (Matsumoto et al., 2013). For therapy monitoring, dynamic changes in EV-derived ncRNAs can reflect pharmacological responses. For example, upregulation of miR-425-5p after SGLT2 inhibitor treatment is associated with metabolic improvement (Park J. H. et al., 2025). Although EVs show promising applications across different stages of HF, the optimal applicability of EVs from different sources in specific HF subtypes remains insufficiently characterized, limiting their clinical stratification value.

### 7.5 Regulatory pathways and quality contro

Compared with well-established small-molecule drugs and biologics, no EV-based therapeutics have yet been formally approved for clinical use, and the global regulatory framework for EVs is still evolving. Regulatory systems differ substantially across regions. Specifically, the FDA primarily classifies EVs-related products under Human Cells, Tissues, and Cellular and Tissue-Based Products or biologics pathways, where some products may be exempt from premarket approval under defined conditions, whereas biologics generally require IND applications and Biologics License Applications. In contrast, the European Union regulates EV-based products under the Advanced Therapy Medicinal Products framework, while Japan classifies them under regenerative medicine-related legislation. Overall, the regulatory status of EVs remains heterogeneous, and clinical development typically relies on IND-supporting studies, including pharmacokinetic, toxicological, and pharmacodynamic evaluations (Wang C. K. et al., 2024).

In terms of manufacturing and quality control, EVs industrialization still faces critical bottlenecks, including insufficient batch-to-batch consistency, complex production processes, and difficulties in large-scale manufacturing (Wiest and Zubair, 2025). Different culture systems, such as microcarrier-based culture and 3D perfusion bioreactors, can significantly influence EVs yield and molecular composition, highlighting the importance of standardized production platforms (Uman et al., 2026). In addition, storage and transport conditions markedly affect EVs stability and biological activity, making optimization essential for maintaining therapeutic efficacy (Qasim et al., 2019). Currently, the MISEV2023 guidelines and Good Manufacturing Practice standards provide fundamental frameworks for EV production, isolation, and characterization, while regulatory agencies in different countries have also issued relevant guidance. However, a globally harmonized regulatory standard and quality evaluation system is still lacking, which continues to hinder the clinical translation and industrialization of EV-based therapies (Terai et al., 2025).

### 7.6 Future directions

In parallel with ongoing efforts to address current challenges in EV-based heart failure research, several emerging directions are expanding the landscape of this field. The development of iPSC-derived cardiac organoids has enabled the generation of multicellular, structurally organized cardiac models that partially recapitulate native myocardial architecture and function (Volmert et al., 2023; Lee et al., 2022). Compared with EVs derived from conventional two-dimensional cultures, cardiac organoid-derived EVs may contain more diverse signaling molecules reflecting multicellular interactions, thereby potentially providing more physiologically relevant disease information and therapeutic cues. Although still at an early translational stage, this platform represents a promising avenue for improving disease modeling, biomarker discovery, and cell-free therapeutic development in HF.

Another exciting frontier lies in plant-derived EVs-like nanoparticles. These nanovesicles, isolated from edible plants such as fruits, vegetables, and herbs, offer advantages including abundant availability, scalability, and low immunogenicity (Zhao et al., 2024). Unlike mammalian cell-derived EVs, plant-derived EV-like nanoparticles possess dual value, their bioactive components can directly exert cardioprotective effects through anti-inflammatory and antioxidant mechanisms, while their excellent gastrointestinal stability provides a unique natural platform for oral delivery in HF (Dad et al., 2021; Cao et al., 2023). Realizing this oral delivery strategy will depend on improving *in vivo* targeting capacity and delivery efficiency.

### 7.7 Clinical translation landscape of EVs-based therapies in HF

HF related EVs-based therapies remain at an early translational stage, with limited cardiovascular clinical trials to date. Encouragingly, the number of clinical trials involving EVs continues to grow. A search of ClinicalTrials.gov (accessed on 13 June 2026) using the terms “exosomes,” “extracellular vesicles,” or “microvesicles” identified more than 500 ongoing clinical studies, highlighting the rapid expansion of EV-based translational research. However, the exact number may vary depending on search strategy, database updates, and inclusion criteria. Importantly, cardiovascular-related studies account for only a minority of these trials, representing approximately 10%–20% of total EV-related clinical studies across different search strategies and classification criteria, indicating that most investigations remain focused on non-cardiovascular indications.

Within the cardiovascular subset, only a limited number of studies have explored exosomes or EVs as biomarkers or therapeutic agents (Table 3), with the majority focusing on diagnostic or prognostic applications and only two registered trials addressing therapeutic interventions (NCT05774509 and NCT05669144). Notably, clinical trials completed in other disease areas, such as BMSC-derived EV infusion for COVID-19-related acute respiratory distress syndrome, have demonstrated acceptable safety profiles and encouraging therapeutic effects, providing an important reference for potential cardiovascular applications (Lightner et al., 2023).

**TABLE 3:** Summary of clinical studies on CVD involving EVs.

NCT number	Study title	Conditions	Interventions	Status	Research type
NCT05774509	Treatment of non-ischemic cardiomyopathies by intravenous extracellular vesicles of cardiovascular progenitor cells	Heart failure with reduced ejection fraction	Biological: extracellular vesicle-enriched secretome of cardiovascular progenitor cells differentiated from induced pluripotent stem cells	Recruiting	therapeutic effect
NCT05669144	Co-transplantation of mesenchymal stem cell derived exosomes and autologous mitochondria for patients candidate for cabg surgery	Myocardial infarction Myocardial ischemia Myocardial stunning	Biological: mitochondria and msc-derived exosomes	Unknown status *	
NCT06408961	EVOC - EVs in obesity and cardiometabolic disease	Cardiovascular diseases	-	Active, not recruiting	exploring biomarkers
NCT03674255	Echocardiography: value and accuracy at rest and stress	Coronary artery disease Ischaemic heart disease Angina pectoris	-	Enrolling by invitation	
NCT06434207	Extracellular vesicle micro rna profiling in congenital heart disease: fetal-maternal regulation in neonatal thrombosis	Congenital heart disease Single-ventricle Thrombosis	-	Recruiting	
NCT06536686	Silent myocardial ischemia in patients undergoing non-oncological abdominal surgeries	Silent myocardial ischemia Acute myocardial infarction	Genetic: silent myocardial ischemia, stemi		
NCT06064123	Biomarkers for diagnosis, prognosis, and targeted therapy after heart transplantation	Heart transplant rejection	Diagnostic test: cell-free DNA		
NCT05411445	Study of role of blood microvescicles and exosomes in patients with graft occlusion after aortocoronary bypass surgery	Coronary artery bypass surgery	-		
NCT06856993	Early evaluation of exosome multiomics and clinical prognosis in patients with sudden death	Sudden cardiac death Exosome multiomics Clinical prognosis	-		
NCT04127591	Differential expression and analysis of peripheral plasma exosome mirna in patients with myocardial infarction	Myocardial infarction	-	Unknown status *	
NCT04921774	Research on patients with heart transplantation	Heart transplant rejection	-		
NCT03984006	Early detection of autoimmune thyroid heart disease via urinary exosomal proteins	Thyroid diseases Heart failure	-	Completed	
NCT03034265	New biomarkers and difficult-to-treat hypertension	Hypertension	-		
NCT05584943	Kinetics of cevs over the 24-h dosing interval after low-dose aspirin administration	Cardiovascular diseases Vesicle	-	Completed	The effect of drugs on EVs
NCT02931045	Antiplatelet therapy effect on extracellular vesicles in acute myocardial infarction (Gasecka et al., 2019)	Myocardial infarction	Drug: ticagrelor Drug: clopidogrel	Completed With results	

## 8 Conclusion

In summary, EVs form a complex intercellular communication network in heart failure through the bioactive molecules they carry, thereby providing a mechanistic framework that integrates inflammation, metabolism, cell death, fibrosis, and vascular remodeling. This systems-level perspective deepens our understanding of HF pathophysiology and highlights the potential of EVs as multi-target regulatory and therapeutic agents. Stem cell-derived EVs exhibit promising myocardial repair effects, while engineered EVs offer versatile platforms for targeted and precision drug delivery. In addition, EV-associated ncRNAs serve as minimally invasive biomarkers with diagnostic and prognostic value, underscoring their translational potential in heart failure management.

Nevertheless, several key challenges remain, including the lack of standardized isolation and characterization methods, heterogeneity of EV populations, and limited understanding of *in vivo* pharmacokinetics and safety profiles. Future research should prioritize the development of standardized production and analytical pipelines, supported by multi-omics technologies and artificial intelligence, to refine mechanistic insights and improve clinical translatability. Collectively, these efforts will facilitate the reliable application of EVs in the precision diagnosis and treatment of HF.

## Glossary

ADSC: Adipose-derived stem cells
AKT: Protein kinase B
AMPK: AMP-activated protein kinase
BMSC: Bone marrow mesenchymal stem cells
BNP: Brain natriuretic peptide
CDC: Cardiosphere-derived cells
CFs: cardiac fibroblasts
CHF: Chronic heart failure
CircRNA: Circular RNA
CVD: Cardiovascular disease
DCM: Dilated cardiomyopathy
DC: differential centrifugation
DUC: differential ultracentrifugation
ECs: Endothelial Cells
EPC: Endothelial progenitor cells
ESC: Embryonic stem cell
ESE: Early sorting endosome
EVs: Extracellular vesicles
Gal-3: Galectin-3
HF: Heart failure
HUC-MSCs: Human umbilical cord mesenchymal stem cells
I/R: Ischemia/reperfusion
ICAM-1: Intercellular adhesion molecule-1
IL: Interleukin
ILV: Intraluminal vesicles
IPSC: Induced pluripotent stem cells
JAK: Janus kinase
LncRNA: Long non-coding RNA
LSE: Late sorting endosomes
MAPK: p38 mitogen-activated protein kinase
MI: Myocardial infarction
MMP: Matrix metallo proteinase
Mscs: Mesenchymal stem cells
mToR: AMPK -mammalian target of rapamycin
MVB: Multivesicular bodies
Mφ: Macrophage
NcRNA: Non-coding RNA
NF-κB: Nuclear factor kappa-B
PTEN: Phosphate and tension homology deleted on chromosome ten
ROS: Reactive oxygen species
SIRT2: Silent information regulator 2 homolog
STAT: Signal transducer and activator of transcription
TLR: Toll-like receptor
TNF-α: Tumor necrosis factor-α
UC: ultracentrifugation isolation
