# Supplementary material for: Extracellular vesicles in heart failure: bridging pathogenic insights, diagnostic utility, and therapeutic applications
Source: Front Pharmacol. 2026 Jul 31;17:1848964. doi: 10.3389/fphar.2026.1848964 (PMC13472912; doi:10.3389/fphar.2026.1848964)
Supplement: Supplementary file 1 [file Table1.docx]

Supplementary Table 1. Summary of experimental evidence for the regulatory role of EVs in the pathological progression of HF

| EVs | Isolation methods / characterization level | Level of experimental evidence | Sample size | Extent of gain/loss | Limitations |
| --- | --- | --- | --- | --- | --- |
| Balancing Inflammatory Responses in HF by EVs | | | | | |
| Circulating EVs from EAM mouse(miR-142)(32) | dC + UC  NTA/ TEM  CD63/TSG101/Alix/Calnexin/ApoA1 | Mouse  CD4⁺ T | Animal (n=6)  Cell (n=6) | Gain: miR-142 mimics  Loss: miR-142 inhibitor/SOCS1 siRNA | ①Cellular origin of EVs not identified  ②Mechanism of selective miR-142 loading remains unclear |
| Cardiac EVs induced by myocardial IR (33) | dC + UC  NTA/TEM  CD63/CD9/TSG101/Alix | Mouse  Macrophages | Animal (n=3-12)  Cell (n=3-6) | Gain: miR-155 agomir  Loss: GW4869/miR-155 inhibitor | ①GW4869 may exert its effects through other mechanisms  ②Cellular origin of EVs not clearly distinguished |
| CHF plasma EVs(34) | dC+ UC  NTA(50-200 nm)/TEM  CD9/TSG101 | Patient plasma  THP-1/Raji/HUVEC | Patient (n=20)  Cell (n=3) | Loss: TLR9 inhibitor | ①Single-center, small sample size  ②Specific cellular origin of EVs not clarified  ③Validated only in vitro |
| M2-EVs(35). | dC + UC + filtration  TEM(75-82nm)/NanoFCM  CD9/CD63/Alix/Calnexin | Pig/Rat/Mouse  BMDM/RAW264.7/HCAEC | Rat (n=5-10)  Pig (n=3)  Cell (n=3-6) | Gain:oe-CCR2 Loss:miR-181b-5p inhibitor /si-CCR2 | ①Synergistic effects of other miRNAs/proteins not clarified  ②Differences between allogeneic and autologous sources not compared |
| EVs-CDC/IFNγ/EVs-CDC(36) | dUC+filtration, NanoFCM , proteomics-based characterization | Pig  CDCs/PBLs | n=4 | - | ①In vitro experiments only  ②Efficacy of EVs-CDC not validated in other models  ③Functional experiments not performed |
| CDC-EVs(37) | ultrafiltration + PEG precipitation + filtration  NTA(150nm)/TEM/DLS  CD63/Alix/HSP70 | Rat/Pig  Macrophages | Pig (n=4-5)  Rat (n=4-8)  Cell (n=3-4) | Gain:miR-181b mimic  Loss:miR-181b antagomir/PKCδ siRNA | ①miR-181b alone is less effective than intact EVs, suggesting synergy with other components  ②Downstream mechanisms of PKCδ not fully elucidated |
| CDC-EVs(38) | dC + ultrafiltration  NTA(30-150 nm)/TEM | Rat/Mouse  Macrophages | Animal (n=3-8)  Cell (n=3-4) | Gain:miR-26a mimic  Loss:MerTK KO小鼠/anti-C1qa | ①Whether miR-26a alone fully recapitulates the function of CDC-EVs remains unclear  ②Upstream regulatory mechanisms of C1qa not elucidated |
| EMP-EVs(39) | dC | Patient plasma  Mouse  HCAEC | Animal (n=8)  Cell (n=4-10)  Patient (n=20-60) | Loss:miR-222 inhibitor | ①Small clinical sample size  ②Mechanisms underlying miR-222 reduction under high-glucose conditions not elucidated  ③Synergistic effects of other miRNA molecules not excluded |
| 内皮细胞来源EVs(含miR-126)(40) | dC+ExoQuick precipitation+RNA extraction by TRIzol; | Mouse  HUVEC | Animal (n=6-16)  Cell (n=3-6) | Gain:Ad-HSPA12B/miR-126 mimic  Loss:HSPA12B-/-mouse/miR-126 mimics | ①Upstream mechanisms of HSPA12B-mediated miR-126 upregulation not elucidated  ②Cellular source of EVs not clearly defined |
| M2巨噬细胞外泌体(THP1-IL4 exo)(41) | dUC + filtration+  NTA/TEM | Mouse  BMDMs | Animal (n=8-10)  Cell (n=3-4) | Loss:IFNAR neutralizing antibody | ①miR-23a/b-mediated inhibition of cGAS-STING not validated by functional rescue experiments  ②Which specific component directly targets STAT1/IFNAR1 not clarified |
| MSC-EVs(42) | dC+ filtration +TFF  NTA | Rat  MHC-II/APCs | Animal (n=4-8)  Cell (n=4) | Loss:Foxo3 siRNA/LB100､抗CD25/Treg depletion/中和抗IL-10/IL-33/IL-34 | ①Upstream mechanisms of PP2A activation not elucidated  ②APC-dominated Treg cell subset induction not fully characterized |
| MSC-EVs (43) | dUC  NTA (50–150nm)/TEM  CD/CD63/TSG101/Alix. | Rat  RAW264.7 | Animal (n=5-8)  Cell (n=5-6) | Gain:miR-182 mimic  Loss:miR-182 inhibitor/ Clodronate-mediated macrophage depletion /TLR4 KO mouse | ①Targeting of TLR4 by miR-182 not definitively validated  ②Specific mechanisms of crosstalk between PI3K/Akt and NF-κB not elucidated  ③Long-term cardiac function follow-up only 3 weeks |
| MSC^NIC-exo^(44) | dUC  NTA(120–150 nm)/TEM  Alix/Tsg101 | Rat  BMDM | Animal (n=5-10)  Cell (n=3-6) | Gain:miR-125a-5p mimic  Loss:miR-125a-5p inhibitor/si-TRAF6 | ①Uptake of exosomes by cardiomyocytes/fibroblasts not investigated  ②Other differentially expressed miRNAs not investigated  ③Efficacy of coronary/intravenous administration not evaluated |
| UMSC-EVs(miR-24-3p)(45) | dC + UC  NTA/TEM  CD63/TSG101 | Mouse  RAW264.7 | Animal (n=4-5)  Cell (n=3) | Gain: miR-24-3p mimic  Loss:miR-24-3p inhibitor/ Cl₂MDP-mediated macrophage depletion /Exosome freeze-thaw/RNase treatment | ①Whether Plcb3 is the sole direct target of miR-24-3p not excluded  ②Other miRNAs/components may have synergistic effects |
| BMSC-EVs(miR-25-3p(46) | dUC  NTA(130–150 nm)/TEM  CD63/CD9 | Rat  H9C2/RAW264.7 | Animal (n=10)  Cell (n=4) | Gain:Electroporation loading of BMSC-Exo-25-3p | ①Preliminary observation only, requiring independent replication and mechanistic validation  ②Effects of miR-25-3p on in situ cardiac injury not assessed |
| MSC^ATV-EV^(miR-139-3p)(47) | dUC  TEM (100–200nm)  CD63/CD81/TSG101/Alix | Rat  BMDM | Animal (n=5-10)  Cell (n=3-4) | Gain:miR-139-3p mimic  Loss:miR-139-3p inhibitor | ①miR-139-3p may have other target genes  ②Contributions of other differentially expressed miRNAs not clarified |
| CBSC-EVs(miR-182/183)(49) | dUC+ sucrose gradient + filtration  NTA(50–150 nm)/TEM  CD9/CD63 | Mouse  BMDM | Animal (n=6)  Cell (n=3-6) | Gain:miR-182/183 mimics  Loss: miR-182/183 nhibitor | ①Other target genes of miR-182/183 not clarified  ②Contributions of other differentially expressed miRNAs not clarified |
| Modulation of Mitochondrial Homeostasis in HF by EVs | | | | | |
| Liver EVs(miR-122)(50) | UC | Mouse  CM/H9C2  Patient plasma | Animal (n=5-7)  Cell (n=4-5)  Patient (n=23/29) | Gain:miR-122 mimic  Loss:miR-122 sponge | ①Specific downstream pathways not elucidated  ②Whether miR-122 secreted by other metabolically active tissues may also enter the heart and exert additive or synergistic effects not explored |
| Nec-1-Exos(52). | dUC  TEM/DLS(30-200nm)  CD9/CD63/TSG101/Calnexin | Rat  CM | Animal (n=10)  Cell (n=3) | Gain:oe-PARP1/oe-AIFM1 | ①Other components may have synergistic effects  ②Downstream signaling of PARP1/AIFM1 not fully elucidated |
| huMSC-EVs(PINK1 mRNA)(203). | dC  NTA/TEM  CD81/Alix | Mouse  ACM/AC16 | Animal (n=6)  Cell (n=3-4) | Gain:oe-PINK1  Loss:si-PINK1 | Whether PINK1 directly regulates NCLX not confirmed (indirectly via PKA) |
| TSC-EVs  (53). | dUC + filtratio  NTA (113nm)/TEM  CD9/CD63/TGD101 | Mouse  H9C2 | Animal (n=6)  Cell (n=5) | - | ①Loss-of-function experiments not performed  ②Upstream mechanisms of Mfn2 not elucidated  ③Specific active components not validated |
| EPC-EVs(miR-210)(54). | dUC + filtration  NTA(50–150 nm)  CD63 | EC | n=4-6 | Gain:miR-210 mimic | ①In vitro experiments only  ②Loss-of-function experiments not performed  ③Target genes of miR-210 not validated |
| ADSC-EVs(miR-221/222)(55). | dC+ExoQuick-TC precipitation  NTA(122 nm)/TEM  CD9/CD63 | Mouse  H9C2 | Animal (n=5-6)  Cell (n=3-7) | Gain:mimic/TG  Loss:inhibitor/KO | Mechanisms regulating ROS and mitochondrial fission not fully elucidated |
| BMSC-EVs(miR-29c)(56) | dUC  CD9/CD63/Alix | Mouse  NRCM | Animal (n=5)  Cell(n=3) | Gain:miR-29c mimic  Loss:miR-29c inhibitor | Synergistic effects of other miRNAs/components not excluded  Evidence for the causal chain remains incomplete |
| Control of Cardiomyocyte Apoptosis in HF by EVs | | | | | |
| Serum-derived EVs from HF patients / Cardiomyocyte-derived EVs (miR-22-3p)(57). | dUC  TEM  Tsg101/CD63 | Rat  H9c2 | Animal (n=10)  Cell (n=3) | Gain:miR-22-3p agomir/oe-URIN  Loss:miR-22-3p antagomir | ①Direct regulatory relationship between FURIN and apoptosis-related genes not elucidated  ②Small clinical sample size, used only for screening and not validated  ③Differences between doxorubicin-induced model and ischemic HF should be noted |
| Hypoxic CM-EVs(lncRNA AK139128)(58) | UC + filtration  NTA(100–200 nm)/ TEM  CD63/Hsp70/Hsp90/TSG101 | Rat  CFs | Animal (n=3)  Cell (n=3) | Loss:si-AK139128 | ①Downstream target genes of AK139128 not identified  ②Only cellular level assessed in vivo; cardiac function and histology not examined |
| ADSC-EVs(60). | Exo extraction reagent precipitation + centrifugation  TEM  CD63/Calnexin | Rat | Animal (n=10) | - | ①Loss-of-function experiments not performed  ②Specific active components not identified  ③Mechanistic depth limited to detection of apoptosis-related proteins |
| MSC-EVs(miR-129-5p)(61). | dUC  TEM (30–140 nm)  CD81/ TSG101. | Mouse  HL-1 | Animal (n=3-6)  Cell (n=3) | Gain:miR-129-5p mimic  Loss:miR-129-5p inhibitor/si-TRAF3 | ①Other target genes of miR-129-5p may exist  ②Effects of miR-129-5p on glucose metabolism during HF development not explored |
| BMSC-EVs(miR-30e)(62) | dUC  NTA(44–107 nm)/TEM  CD9/CD63/CD81/Alix. | Rat  H9C2 | Animal (n=6)  Cell (n=3) | Gain:oe-LOC/oe-miR-30e | ①Overexpression validation only  ②Direct targeting of LOX1 by miR-30e not validated  ③Upstream-downstream relationship of the NF-κB p65/Caspase-9 pathway not fully elucidated |
| BMSC-EVs(lncRNA GAS5)(63). | UC  TEM/NanoFCM (36–140 nm)  CD63/CD81 | Rat  H9C2 | Animal (n=3)  Cell (n=3) | Gain:oe-GAS5  Loss:sh-UL3 | ① Targeting of GAS5 not clearly defined  ②Regulation of the Hippo pathway by UL3 not elucidated |
| PPY-CHI/hEMSC-Exo(64). | UC  NTA (130nm)/TEM  CD63/CD81/TSG101 | Rat  H9C2/HUVEC | Animal (n=6)  Cell (n=3-6) | Loss: EGFR inhibitor | ①EGFR inhibitor partially reversed the effects, suggesting the possible involvement of other pathways  ②Effects of PPY-CHI itself in the hydrogel not distinguished |
| Shaping Angiogenesis in HF by EVs | | | | | |
| Patient-derived EVs from HF patientss(miR-21-5p)(65). | UC  NTA/TEM(<200 nm)  CD81/Alix/TSG101 | Patient plasma Mouse  NRCM/HUVEC/CFs | Animal (n=6)  Cell (n=3)  patients(n=3) | Gain:miR-21-5p mimic  Loss:anti-miR-21-5p/GW4869 | ①Effects of PPY-CHI itself in the hydrogel not distinguished  ②Specific binding sites of miR-21-5p on PTEN/PDCD4 not validated |
| M1-EVs(miR-155)(66). | dUC + filtration  NT (30–150 nm)/ TEM  Alix/CD63/CD81/Calnexin | Mouse  HCAEC/THP-1/RAW264.7/PM/BMDM | Animal (n=5-8)  Cell (n=3-7) | Gain:miR-155 mimic/LV-miR-155  Loss:anti-miR-155/LV-anti-miR-155/Rab27a KO/aSMase KO; | ①Target genes of miR-155 require further validation  ②Synergistic effects of other miRNAs/proteins not excluded |
| CFs-EVs(miR-200a-3p)(67) | UC  NTA(90 nm)/ TEM | NRVF/EC | n=4-6 | Loss:anti-miR-200a-3p | In vitro cell experiments only |
| CDC-EVs(miR-146a)(69). | Exoquick precipitation + centrifugation  TEM  CD63 | Mouse  NRCM/HUVEC/CFs | Animal (n=3-8)  Cell (n=4-8) | Gain: miR-146a mimic  Loss:miR-146a inhibitor/miR-146a KO小鼠/GW4869; | Validation of miR-146a target genes not performed in primary cells |
| hucMSC-EVs(miR-1246)(70). | dUC  NTA(30–150 nm)/TEM  CD63/TSG101/ALIX/Calnexin | Rat  H9C2/HUVEC | Animal (n=5)  Cell (n=3) | Loss:miR-1246 inhibitor | PRSS23–Snail/α-SMA axis lacks direct functional validation |
| ESC-EVs(71) | UC  qNano (50–125 nm)/ TEM  CD9/Alix | Rat  HUVEC | Animal (n=5-8)  Cell (n=3-6) | Loss:FGF2R siRNA/AZD4547 | Whether FGF2 is the sole key active molecule remains to be fully clarified |
| 3D培养CPC-EVs(72) | UC  TEM | Rat  CPCs | Animal (n=5-8)  Cell (n=3-4) | Gain:-  Loss:DAPT/Notch1-shRNA/GW4869 | ①Specific components not fully clarified  ②Causal relationship between Notch1 and exosome secretion requires further clarification; lacks independent laboratory replication |
| ADSC-EVs(miR-205)(73). | dC+ExoQuick-TC precipitation +  NTA(100nm)/TEM  CD63/CD9/CD81/TSG101/Alix | Mouse  HMEC-1 | Animal (n=8-10)  Cell (n=3-6) | Gain:miR-205 mimic  Loss:miR-205 inhibitor | ①Direct downstream target genes of miR-205 not fully elucidated  ②Lacks independent laboratory replication  ③miR-205 inhibitor not validated in vivo |
| HXYQR-Exo(74) | UC  NTA(30–150 nm)/TEM  CD9/CD63/TSG101/HSP70/Calnexin | Rat  HUVEC | Animal (n=8-10)  Cell (n=5-12) | - | ①Which specific active component mediate the dominant effect remains unclear  ②Long-term safety and immunogenicity not assessed  ③Lack of validation in clinical patients |
| Regulation of Pathological Cardiac Hypertrophy in HF by EVs | | | | | |
| CFs-EVs(miR-21*)(75) | Ltracentrifugation  TEM (50–100 nm)  CD63 | Mouse  CF/NRCM | Animal (n=6-10)  Cell (n=3-6) | gain:miR-21* mimic  loss:miR-21* antagomir/ Pharmacological inhibition | Whether there are other key targets besides PDLIM5 and SORBS2 remains to be fully clarified |
| CFs-EVs(miR-27a*)(76) | filtration + UC  TEM(100nm)  CD63/TSG101/CD9/CD81/HSC70 | Rat  CM | Animal (n=6-8)  Cell (n=3-6) | Gain:miR-27a* mimic  Loss:miR-27a* inhibitor/antagomir | Whether miR-27a* in exosomes is the sole key molecule remains unclear |
| CF-iPSC-EVs(miR-22)(77) | isolation method not detailed  NTA (115 nm) | CFs | Cell (n=5) | - | ①Lack of validation in animal models  ②Gain-of-function/loss-of-function experiments for miR-22 not performed  ③Specific downstream targets and functional mechanisms not systematically validated |
| SFN-CFs-EVs(78) | dUC  CD81/TSG101/Hsp70 | Rat  CMs | Animal (n=6-8)  Cell (n=3-5) | - | ①Specific components not yet clarified  ②Lack of long-term safety and HF preventive/therapeutic effects |
| CMs-EVs(79). | dUC  TEM(200nm)  TSG101 | Mouse  CM/CF | Animal (n=6-10)  Cell (n=3-6) | Loss:Shh Shh inhibitor / Gli1 inhibition / genetic intervention | Whether shh/N-Shh is the sole key cargo remains to be fully clarified |
| Tipifarnib inhibits exosome biogenesis(80). | ExoQuick precipitation  NTA  CD63/CD81/ALIX/FLOT1/ICAM1/EpCam/ANXA5/TSG101/GM130 | Mouse  CM/CF | Animal (n=6-10)  Cell (n=3-6) | Gain:miR 331-5p mimic/oe-HOXC8  Loss:miR 331-5p antagomir/ Knockdown of HOXC8 | The possible involvement of other key EVs besides miR-331-5p has not been excluded |
| TNF-α-CM/CF-EVs(miR-27a/miR-28-3p/miR-34a)(81). | UC  TEM (100 nm)  CD63/TSG101 | Rat  CM/CF | Animal (n=6-8)  Cell (n=3-5) | - | Whether miR-27a/28-3p/34a are the sole active molecules remains unclear |
| CHF-EVs (miR-217)(82). | dUC | Rat  CM/CF | Animal (n=6-10)  Cell (n=3-6) | Gain:miR-217 mimic  Loss:miR-217 inhibitor, si-PTEN | ①Validation in clinical patient samples is limited  ②Specific downstream signaling networks involving PTEN not fully elucidated |
| BMSC-EVs(miR-29a)(84). | dUC +filtration  DLS(30–150 nm)/TEM  CD63/CD9/ALIX/TSG101 | Mouse  CM | Animal (n=6-8)  Cell(n=3-5) | - | Specific downstream targets of miR-29a not experimentally validated |
| MSC-EVs(85). | dUC  NTA (50–150 nm)  CD63 | H9C2 | Cell (n=3-6) | - | Specific components of EVs not fully clarified and not further validated in vivo |
| Regulation of Cardiac Fibrosis in HF by EVs | | | | | |
| Plasma/myocardium miR-208a(86). | UC + ExoQuick precipitation  qNano (50–150 nm)/TEM  CD9/CD63/CD81/Calnexin | Rat  CM | 不明 | gain:oe- miR-208a/oe-Dyrk2  loss:miR-208a antagomir/Dyrk2 siRNA | Single-center  Not replicated in an independent center  Limited sample size |
| DCM-Exos miR-218-5p(88) | UC  TEM(<200 nm)  CD63/CD9/Alix/Calnexin | Mouse  CF | Animal (n=5-6)  Cell (n=3-6) | gain:miR-218-5p mimics  loss:miR-218-5p inhibitor | Single-center  Not replicated in an independent center |
| eWAT-EVs (miR-23a-3p)(89) | UC + filtration  NTA (100–200 nm)/TEM  CD63/CD81/TSG101 | Mouse  CM/CF | Animal (n=5-10)  Cell (n=5-8) | gain:agomiR-23a-3p/miR-23a-3p mimic/oe-RAP1  loss:GW4869/antagomiR-23a-3p/miR-23a-3p inhibitor/siRAP1 | Insufficient long-term follow-up and validation in other cardiac diseases |
| CD4+ T-EVs(miR-142-3p)(90). | dUC  TEM (100nm)  TSG101/CD63/CD81 | Mouse  CF | Animal (n=5)  Cell (n=3-5) | Gain:miR-142-3p mimic  loss:miR-142-3p inhibitor | Potential contributions of other miRNAs not excluded |
| M2 -sEV(circUbe3a)(93) | dUC  TEM  Alix/HSP70/TSG101 | Mouse  CF | Animal (n=3-6)  Cell (n=3) | gain:oe-circUbe3a/miR-138-5p mimic  loss:miR-138-5p inhibitor/circUbe3a siRNA | Some typical fibrotic signaling pathways not addressed |
| CHF-EVs(miR-320a)(94) | ExoRNeasy column purification  NTA(40–200nm)/ TEM  CD63/CD9/TSG101 | Patient plasma  HEH2 | Patient n=10/5  Cell (n=3-6) | Gain:miR-320a mimics  loss:miR-320a inhibitor | ①Some typical fibrotic signaling pathways not addressed  ②Lack of validation in animal modelsLack of validation in animal models |
| Young human plasma sEV(miR-664a-3p)(96). | dUC + filtration  NTA(50–200 nm)/TEM  CD63/CD81/TSG101 | Mouse  CFs | Animal (n=6-8)  Cell (n=3-5) | gain:miR-664a-3p mimics;  loss:miR-664a-3p inhibitor | Lack of validation related to human cardiac fibrosis |
| CBSC-EVs(97) | dUC+filtration +concentration + | Mouse  ARVF/MAAF/NHCF | Animal (n=8-10)  Cell (n=3) | - | ①A single key effector molecule not identified  ②ncRNA mechanisms remain speculative |
| MSC-EVs(98) | UC  NTA (50–100 nm)/TEM  Tsg101/Alix | Rat  CMECs | Animal (n=6)  Cell (n=3) | Loss:GW4869 | ①Specific components not identified  ②PDGFR-β is only a correlative mechanism, with insufficient causal validation |
| oe-circ_0036176 (99) | UC +filtration  NTA (50–150 nm)/TEM  CD63 | Patient plasmaHAFs/mCFs | Patient (n=18-22)  Cell (n=3) | gain:oe-circ_0036176/miR-218-5p mimic  loss:RF mutation (ATG/7nt deletion) | ①Lack of in vivo validation  ②Downstream cyclin/Rb mechanisms require further validation |
